# Heart failure awareness in the Korean general population: Results from the nationwide survey

**DOI:** 10.1371/journal.pone.0222264

**Published:** 2019-09-06

**Authors:** Mi-Hyang Jung, Hack-Lyoung Kim, Jae Hyuk Choi, Sunki Lee, Min Gyu Kong, Jin Oh Na, Yang Hyun Cho, Kyoung-Im Cho, Dong-Ju Choi, Eung Ju Kim

**Affiliations:** 1 Division of Cardiology, Dongtan Sacred Heart Hospital, Hallym University College of Medicine, Gyunggi-do, Republic of Korea; 2 Division of Cardiology, SMG-SNU Boramae Medical Center, Seoul National University College of Medicine, Seoul, Republic of Korea; 3 Division of Cardiology, Hangang Sacred Heart Hospital, Hallym University College of Medicine, Seoul, Republic of Korea; 4 Department of Cardiology, Soon Chun Hyang University Bucheon Hospital, Bucheon, Republic of Korea; 5 Division of Cardiology, Korea University Guro Hospital, Korea University College of Medicine, Seoul, Republic of Korea; 6 Department of Thoracic and Cardiovascular Surgery, Heart Vascular Stroke Institute, Samsung Medical Center, Sungkyunkwan University School of Medicine, Seoul, Republic of Korea; 7 Division of Cardiology, Kosin University Gospel Hospital, Kosin University College of Medicine, Busan, Republic of Korea; 8 Department of Internal Medicine, College of Medicine, Seoul National University Bundang Hospital, Seongnam, Republic of Korea; Kurume University School of Medicine, JAPAN

## Abstract

**Background:**

For a better heart failure outcome, it is fundamental to improve the awareness of heart failure at the general population level. We conducted this study to identify the current status of awareness of heart failure in the Korean general population.

**Methods:**

This cross-sectional nationwide survey recruited a total of 1,032 participants aged 30 years or older, based on a stratification systematic sampling method. A 23-item questionnaire was surveyed through telephone interviews.

**Results:**

Although 80% of the participants had heard of heart failure, 47% exactly defined what heart failure is. A minority of participants correctly recognized the lifetime risk of developing heart failure (21%) as well as the mortality (16%) and readmission risk (18%) of heart failure and the cost burden of heart failure admission (28%). Regarding preferred treatment options, 71% of the participants chose a treatment option that could improve the quality of life. Approximately two-thirds of the participants agreed that current medical treatment could reduce mortality and improve the quality of life. More than half of the participants (59%) thought that heart failure patients should live quietly and reduce all physical activities. Across survey items, we found a lower awareness state in the elderly groups and people at lower income and educational levels.

**Conclusions:**

The current awareness status of heart failure in the Korean general population is still low. Proactive educational efforts should be made to improve public awareness with special attention to individuals with lower disease awareness.

## Introduction

Heart failure (HF) is a growing disease burden worldwide, including Korea, due to increasing elderly population and comorbidity [1–4]. To achieve a better outcome, multifaceted approaches should be investigated to detect both ‘disease’ and ‘out-of-disease’ factors [5]. Despite recent remarkable advances in HF therapy with unravelling of disease pathways, ‘out-of-disease’ factors, such as educational or socio-economic factors, have been relatively unexplored and out of the interest in most research. In this regard, the public awareness of HF is an important issue for controlling the disease burden. Correct recognition of the symptoms, severity, and prognosis of HF enables early access to medical systems and subsequent proper diagnosis and management.

However, limited studies are available regarding the current awareness state of HF at the population level, and most of them are from European countries [5–8]. Previous European survey data have shown that approximately one-third of the participants misrecognized HF as a normal aging process [5–7]. Furthermore, many individuals perceived HF as less severe disease than other chronic diseases such as cancer [5,6]. Unlike continued concerns about cancer, little attention has been paid to HF in the public as well as in health authorities.

We conducted this contemporary population-based survey to identify the current status of awareness and perception of HF in the Korean general population, anticipating that the survey results could serve as a platform for establishing guidelines and health policies that promote appropriate preventive strategies and educational programs.

## Methods

### Study design and data collection

This cross-sectional telephone survey was conducted between September 19 and October 29 of 2018. The survey process, except for the study design, was outsourced to a research company (Megaresearch, Seoul, Korea). The estimated target population (aged ≥30 years) in September 2018 was 34,472,068. To represent Korean adults aged ≥30 years, the survey population (N = 1,000) was selected using a stratification systematic sampling method considering sex, age (30–64 and ≥65 years), and place of residence (7 metropolitan cities, 9 provinces). For telephone interviews, a random digit dialling (RDD) method was introduced to ensure that households in each zone had an equal chance to be contacted. As many of them do not own landline telephones and preferentially use mobile phones, 20% of the total survey sample was carried out by mobile phone RDD [9]. Structured interviews were conducted by well-trained surveyors with an average of 15 years of experience. During the survey, real-time monitoring was conducted for each surveying staff. Five percent of the survey was verified, and in case of inappropriate surveying, the surveyors were re-educated and conducted a new survey after discarding the previous one.

### Study population

Eligible participants were adults aged 30 years or older, agreed to the survey, and could understand and answer the questions. We excluded participants who (1) were aged <30 years, (2) did not agree to participate in the survey, (3) had difficulty in speaking Korean, (4) could not understand the questions, and (5) could not finish all the way through the final questions.

### Questionnaire

A draft questionnaire was developed by the publicity committee of the Korean Society of Heart Failure. The questionnaire items were carefully selected by reviewing/revising the previous surveys [5–8] and also by adding several new questions reflecting local factors. To confirm the eligibility of the study population, questions regarding age, sex, and place of residence preceded the main survey (S1 Table). The basic framework of the questionnaire was similar to that of the previous survey conductedby Remme and his colleagues [5,8]. The main survey was divided into 2 sections (S2 Table). One is a section capturing disease perception before the definition of HF was given and the other is a section capturing the awareness of disease after the definition of HF was given (*e*.*g*., etiology, severity, healthcare cost, prognosis, and treatment). Following the main survey, other demographic factors, such as educational attainment, income, presence of disease (responder, family members), alcohol consumption, and smoking, were also surveyed (S3 Table). Details of questionnaire items are provided in S1–S3 Tables.

### Ethics

The local institutional review board of Korea University Guro Hospital approved this study and waived written informed consent.

### Statistical analysis

Categorical variables are presented as frequency on each questionnaire item. Continuous variables are presented as mean and standard deviation. The chi-square or Fisher’s exact test was performed on each questionnaire item to identify whether the awareness status significantly differed according to demographic characteristics (such as sex, age group, residency, educational attainment, household income, and presence of comorbidity). All analyses were conducted using the statistical program package SPSS (IBM Co., Armonk, NY), version 22 and two-sided *P* values <0.05 were considered statistically significant.

## Results

### Characteristics of the study population

A total of 1,032 participants completed the RDD telephone survey based on stratified system sampling. The study sample was evenly distributed by sex, age, and place of residence. The mean age of the study population was 58.2 ± 14.6 years (ranging from 30 to 92 years). Approximately half of the participants (48.8%) were college graduates, and one-third (34.5%) had cardiovascular comorbidities (hypertension, diabetes, and dyslipidemia). Pre-existing HF was identified by themselves or their family members in 14 participants (4.0%). Details of the study sample are summarized in S4 Table.

### Questions before the definition of HF was given

**Fig 1:**
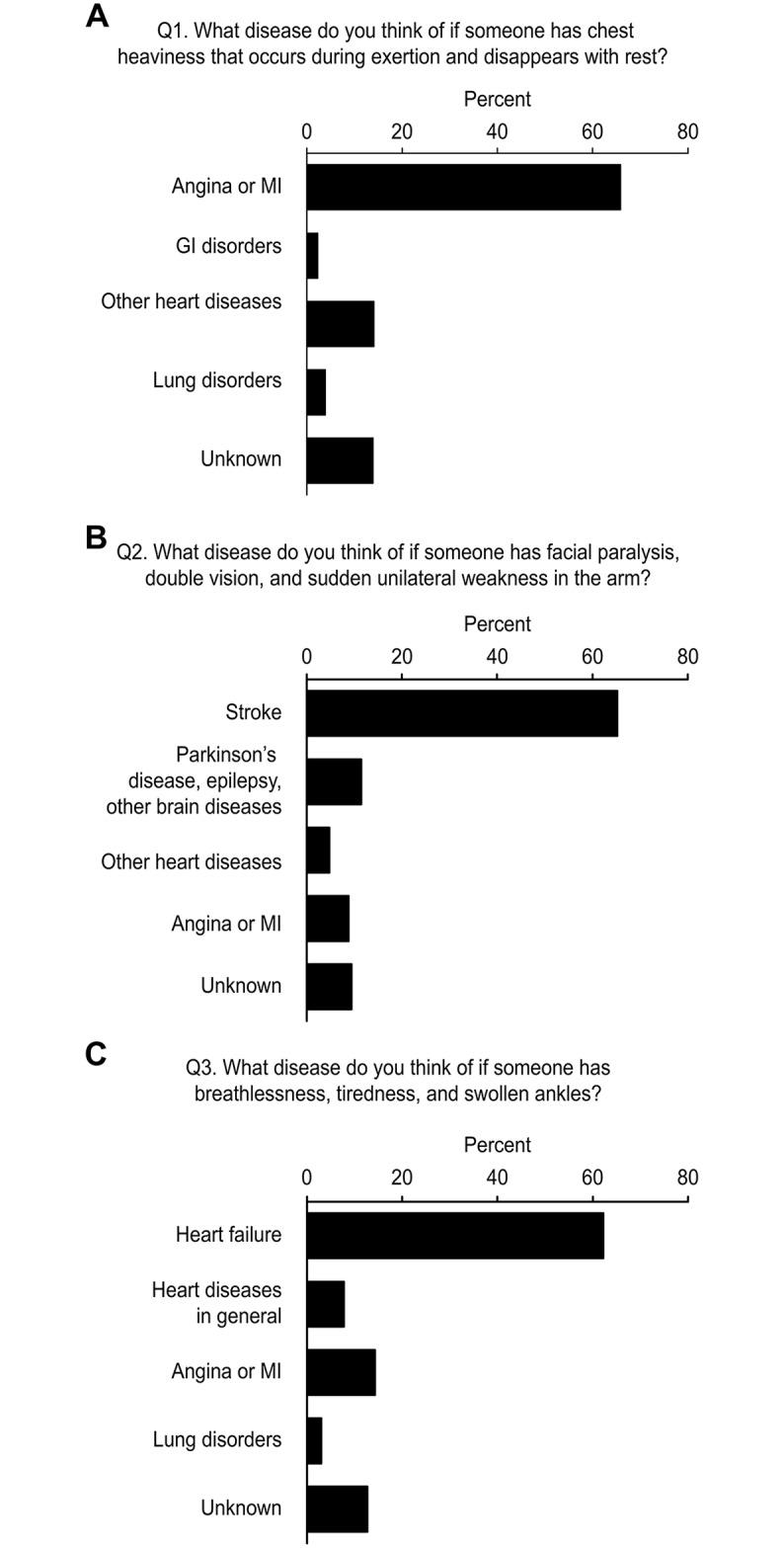
The awareness state of symptoms of 3 representative cardiovascular diseases. A. Question for angina/MI (Q1) B. Question for stroke (Q2) C. Question for heart failure (Q3) GI, gastrointestinal; MI, myocardial infarction.

The first 3 questions identified the awareness of the symptoms of each disease (Fig 1). For the comparison, questions regarding 2 other major cardiovascular diseases (Q1 for angina/myocardial infarction and Q2 for stroke) were also provided. Approximately two-thirds (62%-66%) of the participants answered correctly, but the percentages of correct recognition of HF symptoms were slightly lower than those of the 2 other diseases.

When the participants were asked if they ever had heard of HF, 80.1% answered that they had heard of it. In contrast, when the participants were asked to choose the best description of HF, only 47.3% chose the description ‘the heart cannot pump enough blood around the body’ (S1 Fig).

When the participants were asked about symptom severity involving ‘breathlessness, tiredness, or swollen ankles’, 62.0% recognized that it was a serious illness (S2 Fig). When asked ‘how soon will you go to the hospital if you experience breathlessness, tiredness, or swollen ankles?’, approximately half of the participants (54.9%) answered that they would visit the hospital within 1–2 days, followed by within 1 week (27.7%), 1 month (7.8%), and 1–3 weeks (7.5%); 2.0% answered that they would not seek medical help (S3 Fig). One out of 10 people or their family members had heart disease: angina or myocardial infarction was the most prevalent disease (57.3%), followed by HF (15.5%), valvular heart disease (12.6%), and arrhythmia (8.7%) (S4 Fig).

### Questions after the definition of HF was given

#### Etiology of HF

Approximately one-third of the participants (35.3%) answered that HF was a normal aging process. When the participants were asked about the precipitating factors for HF, 74.1% chose hypertension and 15.4% lung disease (S5 Fig).

#### Perception of HF-related risk

**Fig 2:**
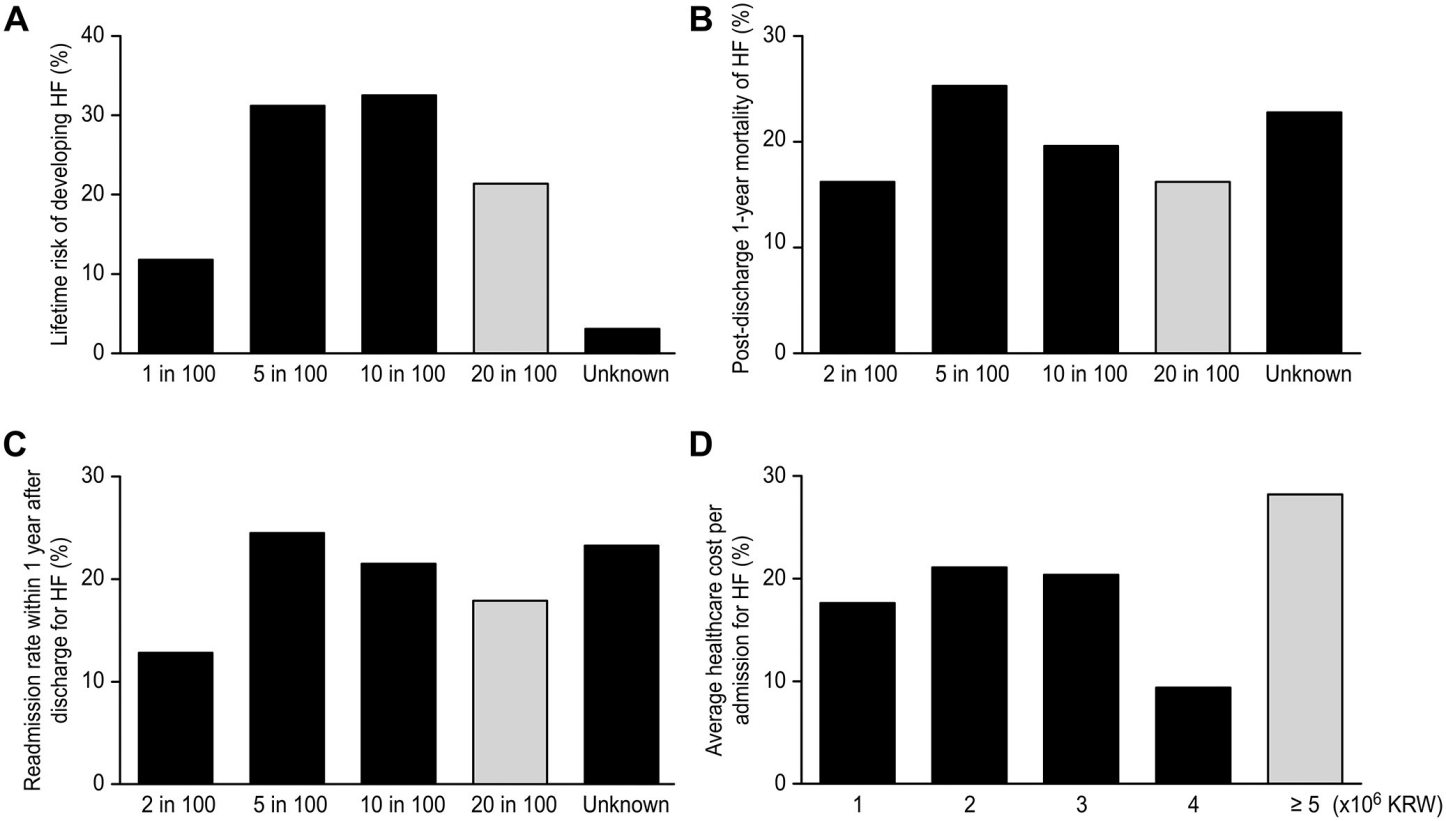
The awareness state of HF in terms of its risk and cost. A. Lifetime risk of developing HF B. Post-discharge 1-year mortality of HF C. Readmission rate within 1 year after discharge for HF D. Average healthcare cost per admission for HF HF; heart failure. The numbers provided on the *x*-axis of the graph are approximate ones expected. The correct answers are presented in gray color.

When the participants were asked about the lifetime risk of developing HF, only 21.4% answered correctly as ‘20 in 100 people’ (Fig 2A). When the participants were asked about ‘the most likely disease that has the highest mortality within 5 years after the diagnosis’, 34.3% chose myocardial infarction, 25.4% HF, 23.0% stroke, and 17.1% prostate or breast cancer (S6 Fig). Regarding their concerns about sudden death risk in HF patients, 62.4% of the participants answered that they were worried about sudden cardiac death; however, 23.6% answered that they were not worried about sudden cardiac death. The perception of acute HF risk (post-discharge 1-year mortality) was also captured (Fig 2B). Surprisingly, only 16.2% of the participants answered correctly as ‘20 in 100 people might die’ and more participants thought that ‘5 in 100 people might die’ (25.3%) or ‘10 in 100 people might die’ (19.6%). When the participants were asked about the readmission rate within 1 year after discharge from HF (Fig 2C), only 17.9% answered correctly as ‘20 in 100 people’. When the average health care cost per admission from acute HF was surveyed, 28.2% answered correctly at 5,000,000 KRW or greater (US $1 = 1113.5 KRW as of October 2018, Fig 2D). When asked about the disease that has the greatest impact on the quality of life among 4 chronic diseases, 36.2% of the participants answered that it is diabetes, followed by HF (33.8%), hypertension (17.8%), arthritis (10.4%), and ‘do not know’ (1.7%) (S7 Fig).

#### Treatment of HF

**Fig 3:**
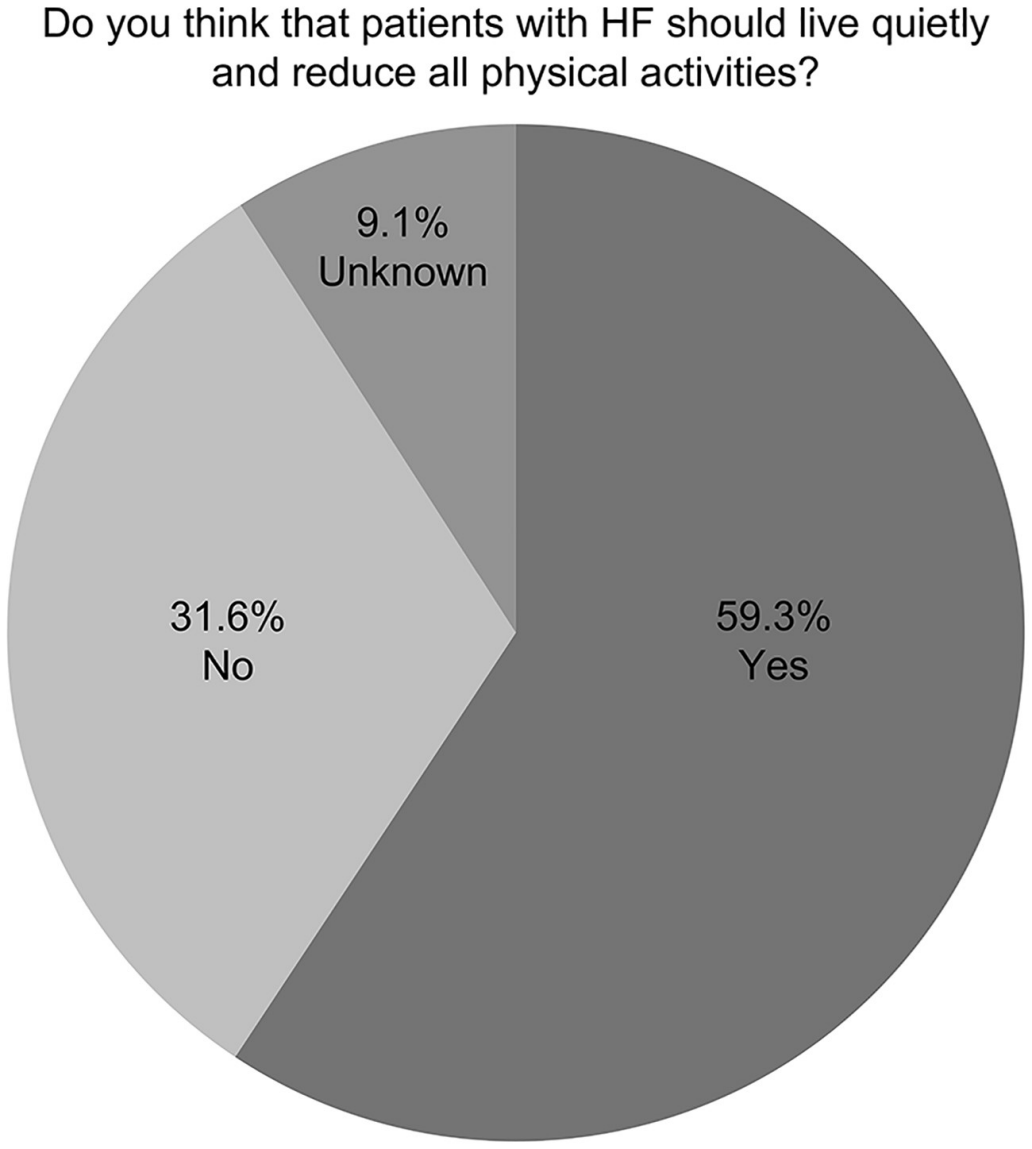
Perception regarding the proper physical activity level in HF. HF; heart failure.

Regarding the preferred treatment option, 71.0% of the participants chose a treatment option that could improve the quality of life, and 17.6% chose a treatment option that could make one to live longer. Their perception of current HF medication was also surveyed. Overall, 62.0% of the participants agreed that ‘current HF medication could reduce deaths from HF’, 66.7% agreed that ‘current HF medication could improve the quality of life’, and 64.9% agreed that ‘current HF medications could prevent the occurrence of HF’. When the participants were asked if patients with HF should live quietly and reduce all physical activities, more than half of the participants (59.3%) answered ‘yes’ (Fig 3).

#### Source of information about HF

For the source of information about HF, 66.9% of the participants answered that they would visit secondary or tertiary care clinics, followed by primary care clinics (19.9%), the internet (11.6%), and rarely oriental medicine clinics (1.0%) or pharmacies (0.7%) (S8 Fig).

#### Awareness of HF based on age, sex, and socioeconomic factors

Table 1 shows the differences in HF symptom awareness among subgroups. Awareness of disease symptoms was significantly poor when the participants (1) were older (≥65years), (2) were living in a rural area (*eup*, *myeon*, *ri*), (3) were at a lower income level, and (4) were at a lower educational level. Awareness of HF symptoms in participants aged 70–79 years was only 49.7% and it was much lower (28.8%) in those aged ≥80 years. Awareness of HF symptoms was significantly lower in participants who had comorbidities (hypertension, diabetes, and dyslipidemia) (55.6%) than in those without (65.7%).

**Table 1:** Differences in the awareness of heart failure symptom among subgroups (N = 1,032).

Q3: What do you think if someone has the following symptoms? Symptoms: breathlessness with low level activity, tiredness, and swollen ankles						
	Answer					
	Heart failure	Heart in general*	Angina or myocardial infarction	Lung disorders	I do not know	*P* value
Total	642 (62.2)	80 (7.8)	148 (14.3)	31 (3.0)	131 (12.7)	-
Sex						0.136
Male	335 (64.2)	33 (6.3)	80 (15.3)	17 (3.3)	57 (10.9)	
Female	307 (60.2)	47 (9.2)	68 (13.3)	14 (2.7)	74 (14.5)	
Age (binary)						<0.001
30–64 years	382 (71.4)	34 (6.4)	72 (13.5)	9 (1.7)	38 (7.1)	
≥65 years	260 (52.3)	46 (9.3)	76 (15.3)	22 (4.4)	93 (18.7)	
Urbanization level of residence						<0.05
Urban (*dong*)	558 (62.9)	68 (7.7)	133 (15.0)	27 (3.0)	101 (11.4)	
Rural (*eup*, *myeon*, *ri*)	84 (57.9)	12 (8.3)	15 (10.3)	4 (2.8)	30 (20.7)	
Educational attainment						<0.001
Middle school or less	82 (39.6)	23 (11.1)	28 (13.5)	11 (11.1)	63 (30.4)	
High school	177 (57.3)	35 (11.3)	53 (17.2)	6 (11.3)	38 (12.3)	
College or more	374 (74.2)	22 (4.4)	66 (13.1)	14 (4.4)	28 (5.6)	
Do not want to say	9 (75.0)	0 (0.0)	1 (8.3)	0 (0.0)	2 (16.7)	
Household income (HI, KRW 1,000^†^)						<0.001
HI ≤ 1,000	36 (41.4)	5 (5.7)	10 (11.5)	3 (3.4)	33 (37.9)	
1,000 < HI ≤ 2,000	62 (55.9)	10 (9.0)	20 (18.0)	3 (2.7)	16 (14.4)	
2,000 < HI ≤ 3,000	146 (58.9)	29 (11.7)	40 (16.1)	9 (3.6)	24 (9.7)	
3,000 < HI ≤ 4,000	140 (61.1)	17 (7.4)	33 (13.1)	9 (3.9)	30 (13.1)	
4,000 < HI ≤ 5,000	113 (72.4)	10 (6.4)	18 (11.5)	4 (2.6)	11 (7.1)	
HI > 5,000	122 (74.4)	6 (3.7)	22 (13.4)	2 (1.2)	12 (7.3)	
Do not want to say	23 (62.2)	3 (8.1)	5 (13.5)	1 (2.7)	5(13.5)	
Presence of comorbidity^‡^						0.020
Yes	198 (55.6)	28 (7.9)	62 (17.4)	14 (3.9)	54 (15.2)	
No	444 (65.7)	52 (7.7)	86 (12.7)	17 (2.5)	77 (11.4)	

Data are expressed as number (percentages).

*This means they think it is related to the heart, but they do not know what the specific problem is (*e*.*g*., myocardial infarction, heart failure, and arrhythmia).

^†^US $1 = 1113.5 Korean won (KRW) as of October 2018.

^‡^Comorbidities (hypertension, diabetes and dyslipidemia) of the respondents were surveyed.

Other data on age, sex, and socioeconomic factors are provided in S5–S23 Tables. Participants–who were elderly (≥65 years), were living in a rural area, were at a lower income level, and had comorbidities–paid earlier visits to the hospital (within 1–2 days) than their counterpart (S8 Table); these results were somewhat different from those of other survey items. For the preferred information route, participants who were younger (<65 years), were at a higher income level, and were at a higher educational level tended to more frequently use the internet (S23 Table).

There was similar awareness of the risk and health care costs of HF between men and women, while there were several differences in the response rates. Compared to men, women were less frequently heard of HF (S5 Table), perceived HF symptoms as less severe (S7 Table), and paid later visits to the hospital when suspect symptoms occurred (S8 Table). Women did not precisely recognize precipitating factor for HF (S10 Table) and less frequently answered that current HF medications could prevent the occurrence of HF (S21 Table). Women more frequently answered that HF patients should reduce their physical activities (S22 Table).

## Discussion

In this survey on the public awareness and perception of HF in the Korean general population, we found that a substantial number of participants underrecognized the symptoms of HF and underestimated the risk of HF. Of note, more than half of the participants had a misconception about proper physical activities of HF patients. Also, our data exhibited differences in awareness status and route of obtaining health information based on various socioeconomic factors, suggesting potential targets and effective strategies for improving awareness status.

### Awareness status of HF from the aspect of its symptoms

Although most participants (80%) had heard of HF, less than half of them (47%) were able to exactly define what HF is, which was lower than the results from the previous Study of Heart Failure Awareness and Perception in Europe (SHAPE) survey (86% and 52%, respectively) [5]. Considering that the SHAPE survey was conducted 16 years ago (in the year 2002), the current awareness state of the Korean population is somewhat disappointing. When the symptoms of HF were given, the percentage of the participants who recognized them correctly as HF was 62%, which was lower compared to ischemic heart disease or stroke, but was much higher compared to the SHAPE survey (3%) [5]. These results indicated the need for more effective educational campaigns to raise HF awareness. Conversely, when the symptoms of HF were provided, approximately 2 out of 3 participants answered that it was a serious illness. Almost half of the participants answered that they would visit the hospital within 1–2 days, which was shorter than the mean elapsed time from symptom recognition to hospital visit (about 7 days) [10,11]. However, Taylor *et al*. [12] have reported that most patients initially recognize their symptoms as normal and sought medical help only when daily activities were impaired. Furthermore, the symptoms of HF cannot be initially recognized by patients because of its insidious onset compared to the acute onset of symptoms in myocardial infarction or stroke.

### Awareness status of HF from the aspect of its etiology

Of note, one-third of the participants had a misconception of HF being a normal aging process, and they may have delayed their hospital visit even when they had suspected HF symptoms. To improve the outcome of HF, they should be informed that symptoms such as dyspnea occurring with low-level activities, swollen ankles, and/or tiredness are not normal physiological processes according to aging and might rather be ‘pathological’ conditions that have high morbidity and mortality.

### Awareness status of HF from the aspect of its risk and cost

In general, the perception of HF ‘risk’ and ‘cost burden’ was disappointingly low. In most questionnaires, the correct answer rate was below 30%. According to the Framingham Heart Study, the lifetime risk of developing HF was 20% regardless of sex [13]. However, only 1 of 5 participants correctly recognized the risk. As for diseases that have the worst long-term prognosis, many Koreans regarded myocardial infarction as a disease with the worst 5-year prognosis. In the literature, the 5-year mortality rate of myocardial infarction has been decreasing and it is currently reported approximately at 33%-44% [14,15]. Conversely, the 5-year mortality of HF has been stationary; it is reported at up to 48.5% according to the UK primary care records [16] and to be even worse (75%) according to the US Medicare data [17]. In Scotland, HF has a worse survival rate than myocardial infarction for both sexes [18]. HF is also known to have poorer outcomes than malignant diseases except for lung cancer [18,19]. Unlike the previous SHAPE study [5], the question of our study was restricted to prostate or breast cancer, so that our results are somewhat different from those of the SHAPE study (overall malignancies).

Although recent HF medications and devices have been reducing sudden cardiac death risk, it is important to recognize that HF can possibly entail sudden cardiac death risk. However, up to 38% of the participants did not recognize the risk of sudden cardiac death from HF. Furthermore, the prognosis of HF is quite poor. According to the results from the Korean Acute Heart Failure Registry, 1-year mortality and 1-year rehospitalization rates after hospital discharge were approximately 1 in 5 (18.2% and 23.1%, respectively) [20]. However, only less than 20% of the participants were aware of the actual risk.

The health care burden (medical bills that include both out-of-pocket and insurance copayments) per admission for acute HF was greater than KRW 5,000,000 (US $1 = 1113.5 KRW as of October 2018) [21]. However, more than two-thirds of the participants did not know the exact health care cost.

It is well documented that patients with HF have a significant impairment in the quality of life from the aspects of both physical and mental health, and that HF has more adverse effects as compared to other chronic diseases [22,23]. Unfortunately, in the current survey, almost 66% of the participants thought that HF less affected the quality of life than other clinical disease entities. Better understanding the true risk and health care burden of HF is the first step in early diagnosis/treatment and establishment of appropriate management strategies in the general population.

### Awareness status of HF from the aspect of its treatment

Most participants chose a treatment option that could improve the quality of life. This preference underscores the importance of symptom management that enables their daily life activities. In regard to the perception of the benefit of current HF medications, two-thirds of the participants had a positive response. Unexpectedly, more than half of the participants thought that HF patients should live quietly and reduce all physical activities. Recent studies have demonstrated that exercise improves the quality of life as well as clinical outcomes in stable HF patients [24, 25]. A previous study intriguingly showed that the barrier to the performance of physical activities in HF patients was related to the level of education, exercise self-efficacy, and motivation, but not to the severity of symptoms [26]. More proactive efforts are needed for both patients and clinicians to perform exercise and/or physical activities in appropriate frequency, intensity and mode.

### Preferred information sources

Almost two-thirds of the participants answered that the secondary and tertiary care clinics were the preferred source for health information regardless of age. When the secondary and tertiary care clinics were excluded, elderly participants preferred primary care clinics, whereas younger adults preferred the internet. Considering that HF is highly prevalent in the elderly, it might be more effective in informing the general physician about the mortality and morbidity of HF as well as in timely referring suspected patients [27]. In this regard, surveying the awareness state of the physician might also be helpful. Furthermore, the use of the internet has been increasing over time according to recent survey studies [28]. The internet can be a more effective tool for sharing health information.

### Differential awareness status of HF based on age, sex, and socioeconomic factors

Generally, disease awareness status was lower in the elderly, who were at high risk of developing HF, and was also lower when they had a lower income or educational status. Lower disease awareness might partially explain the higher incidence and earlier development of HF in socioeconomically deprived subjects [2]. Collectively, the current study highlights the need for a systematic approach (*e*.*g*., community support, policy framework, and educational campaign) to improve the awareness status and ultimately clinical outcomes, particularly targeting those who were elderly as well as people at lower income and educational levels [29].

In the current survey, women had a similar awareness status with regard to the risk and health care costs of HF compared to men; however, women tended to recognize that HF symptoms were less severe and did not choose quicker medical treatment, which might have affected clinical outcomes [30, 31].

### Strengths and limitations of our study

The present study has several strengths. The study sample was a representative Korean cohort in terms of age, sex, and residency. Furthermore, telephone interviews were performed by a trained staff member. To the best of our knowledge, this was the first report of public awareness of HF in the Asian population. Our study was the first population-based awareness survey describing differences in the awareness of HF regarding various sociodemograghic factors. It could help establish appropriate health educational programs by identifying vulnerable groups that need more attention.

There are some limitations to our study. First, selection bias might have been caused, because we only included participants who completed the whole questionnaire. These participants might have been concerned about health and could have understood almost all of the questions. Thus, the awareness status in real-world practice might be much lower. Secondly, some of the survey items (e.g., lifetime risk of developing HF) were not based on Korean data. Thirdly, the mode of survey might have been less ideal. Although face-to-face survey is known to be the best method, we could not perform face-to-face survey with better representing of the general population, due to limited cost and time. Finally, data might have been biased toward socially acceptable responses as opposed to their true perception. Despite these limitations, the present study revealed that the status of HF awareness is still low at the present time.

## Conclusions

In this survey on the public awareness and perception of HF in the Korean general population, it was found that a substantial number of participants underrecognized the symptoms of HF and underestimated the risk of HF. Differences in awareness status based on various socioeconomic factors highlighted the need for a systematic approach in the elderly groups as well as in people at lower income and educational levels. Educational, social, and policy measures should be developed to improve the awareness and outcome of HF in the general population.

## Supporting information

S1 Fig Words that best describe heart failure. (PDF)

S2 Fig The perception of symptom severity for breathlessness, tiredness, or swollen ankles. (PDF)

S3 Fig Response to the question, ‘how soon will you go to the hospital if you experience breathlessness, tiredness, or swollen ankles?’. (PDF)

S4 Fig Known heart diseases of participants or their family members. (PDF)

S5 Fig Precipitating factors for heart failure. (PDF)

S6 Fig Response to the question, ‘most likely disease that has the highest mortality within 5 years after the diagnosis’. (PDF)

S7 Fig Perception regarding the disease that has the greatest impact on the quality of life. (PDF)

S8 Fig Source of information about heart failure. (PDF)

S1 Table Questionnaire items for identifying eligibility of the study population. (PDF)

S2 Table Main questionnaire contents. (PDF)

S3 Table Questionnaire items for identifying demographic characteristics. (PDF)

S4 Table Clinical and demographic characteristics of the study population (N = 1,032). (PDF)

S5 Table Differences in the awareness of heart failure symptoms among subgroups (Q4). (PDF)

S6 Table Differences in the awareness of heart failure symptoms among subgroups (Q5). (PDF)

S7 Table Differences in the awareness of heart failure symptoms among subgroups (Q6). (PDF)

S8 Table Differences in the awareness of heart failure symptoms among subgroups (Q7). (PDF)

S9 Table Differences in the awareness of heart failure symptoms among subgroups (Q9). (PDF)

S10 Table Differences in the awareness of heart failure symptoms among subgroups (Q10). (PDF)

S11 Table Differences in the awareness of heart failure symptoms among subgroups (Q11). (PDF)

S12 Table Differences in the awareness of heart failure symptoms among subgroups (Q12). (PDF)

S13 Table Differences in the awareness of heart failure symptoms among subgroups (Q13). (PDF)

S14 Table Differences in the awareness of heart failure symptoms among subgroups (Q14). (PDF)

S15 Table Differences in the awareness of heart failure symptoms among subgroups (Q15). (PDF)

S16 Table Differences in the awareness of heart failure symptoms among subgroups (Q16). (PDF)

S17 Table Differences in the awareness of heart failure symptoms among subgroups (Q17). (PDF)

S18 Table Differences in the awareness of heart failure symptoms among subgroups (Q18). (PDF)

S19 Table Differences in the awareness of heart failure symptoms among subgroups (Q19). (PDF)

S20 Table Differences in the awareness of heart failure symptoms among subgroups (Q20). (PDF)

S21 Table Differences in the awareness of heart failure symptoms among subgroups (Q21). (PDF)

S22 Table Differences in the awareness of heart failure symptoms among subgroups (Q22). (PDF)

S23 Table Differences in the awareness of heart failure symptoms among subgroups (Q23). (PDF)
