# Supplementary figures and images for: Heart failure awareness in the Korean general population: Results from the nationwide survey
Source: PLoS One. 2019 Sep 6;14(9):e0222264. doi: 10.1371/journal.pone.0222264 (PMC6731018; doi:10.1371/journal.pone.0222264)

**S1 Fig. Words that best describe heart failure**

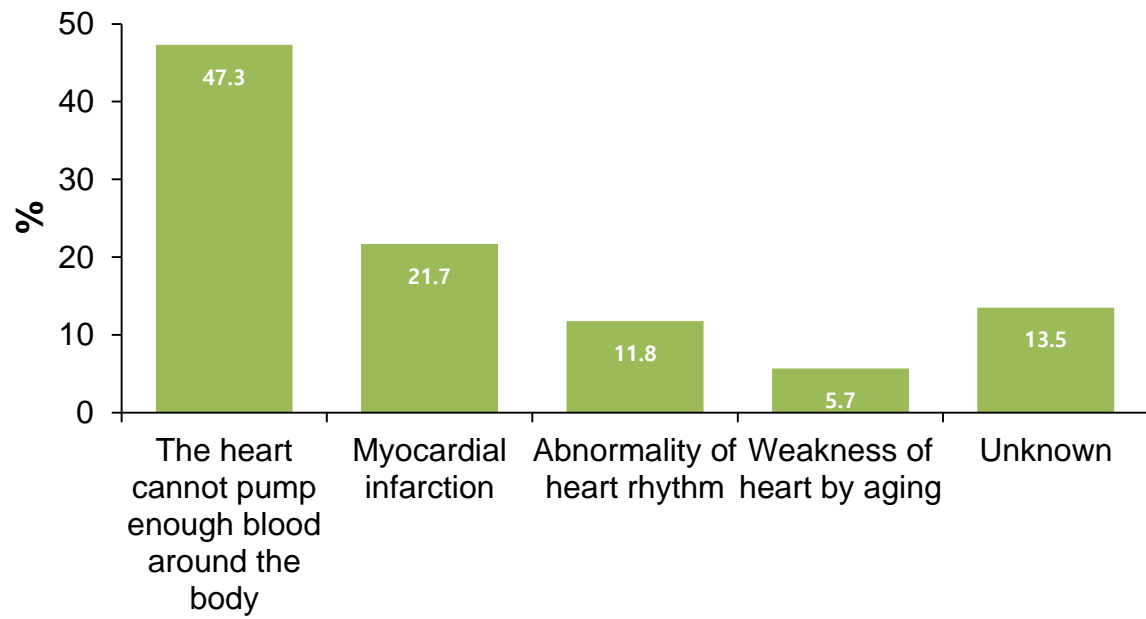

Supplement: S1 Fig — (PDF) [file pone.0222264.s001.pdf]

**S2 Fig. The perception of symptom severity for breathlessness, tiredness, or swollen ankles**

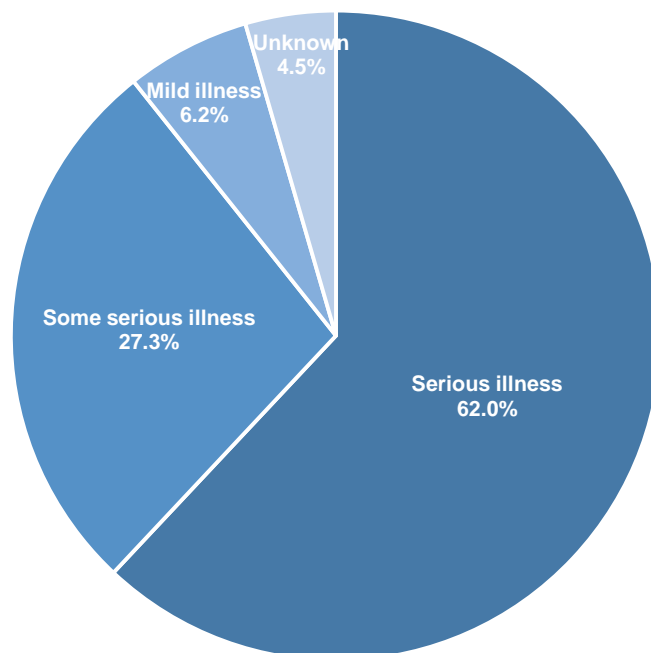

Supplement: S2 Fig — (PDF) [file pone.0222264.s002.pdf]

S4 Fig. Known heart diseases of participants or their family members

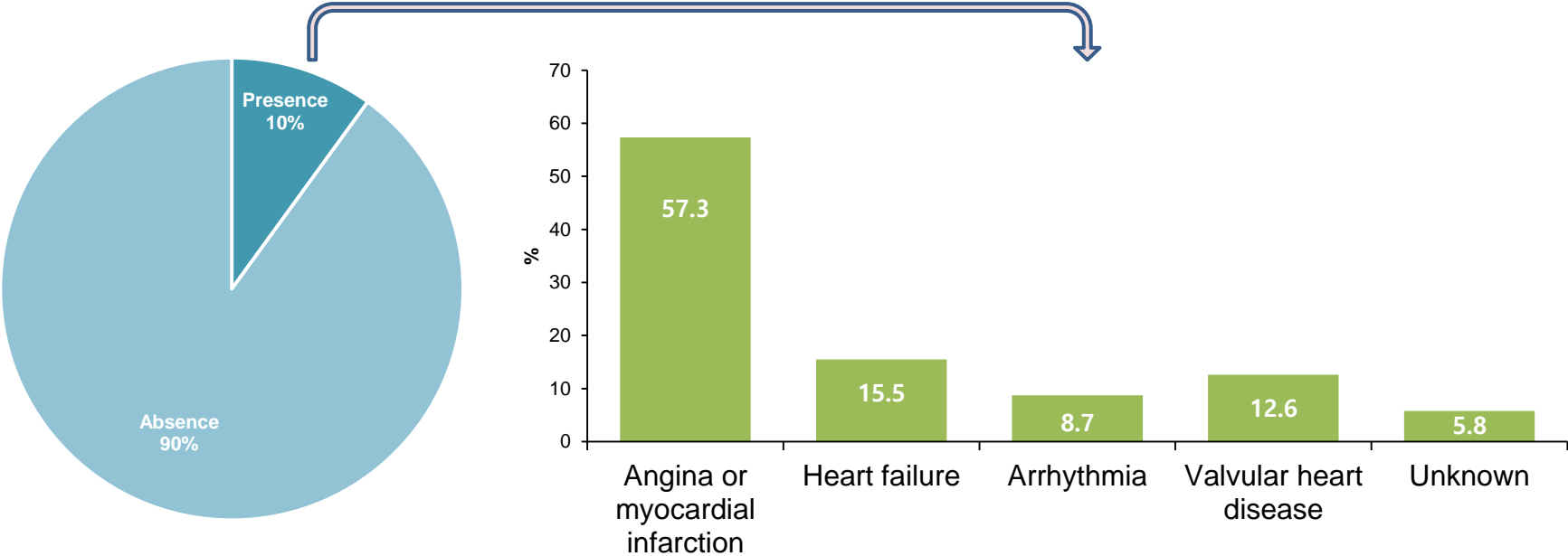

Supplement: S4 Fig — (PDF) [file pone.0222264.s004.pdf]

**S5 Fig. Precipitating factors for heart failure**

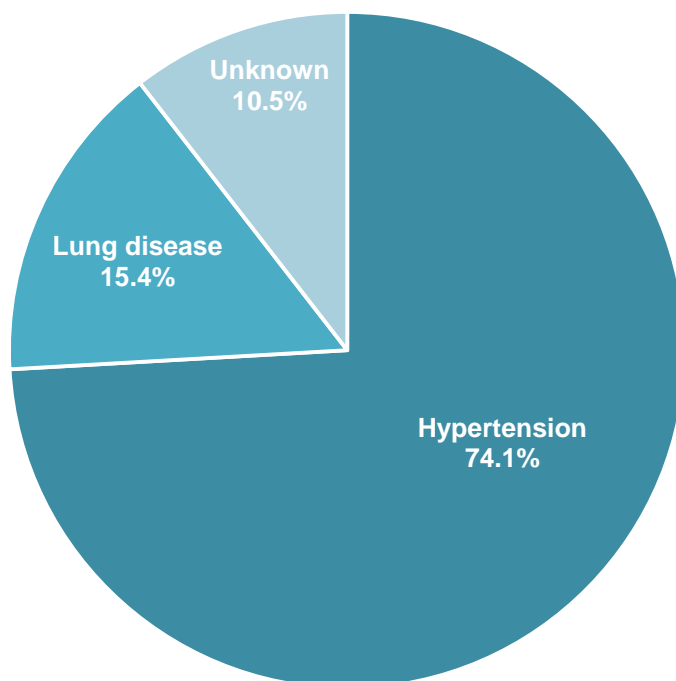

Supplement: S5 Fig — (PDF) [file pone.0222264.s005.pdf]

**S7 Fig. Perception regarding the disease that has the greatest impact on the quality of life**

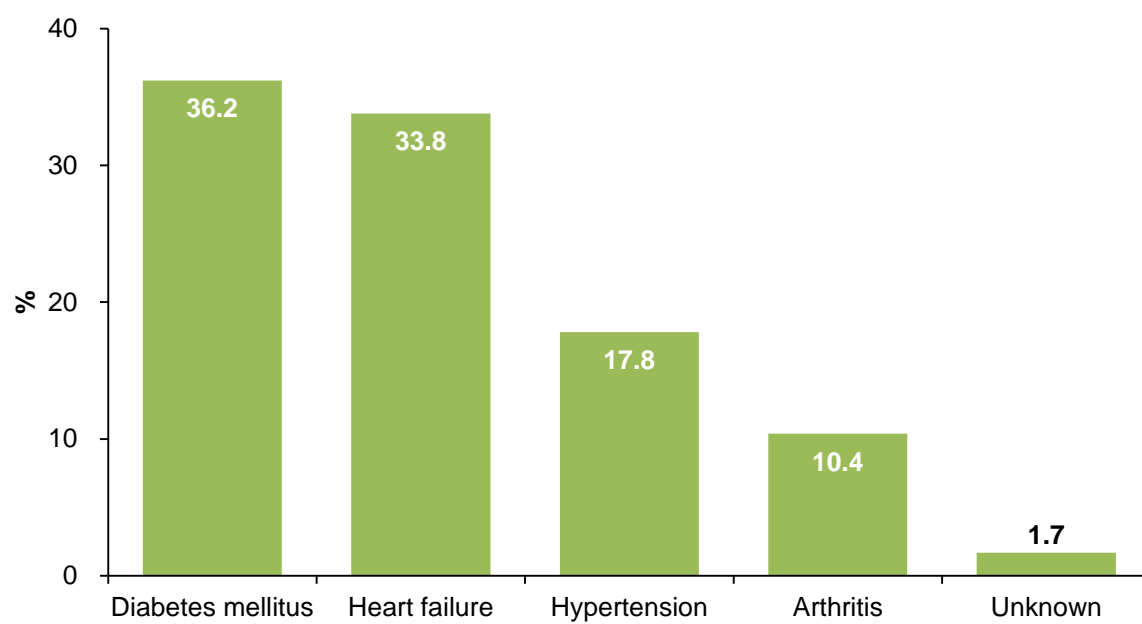

Supplement: S7 Fig — (PDF) [file pone.0222264.s007.pdf]

**S8 Fig. Source of information about heart failure**

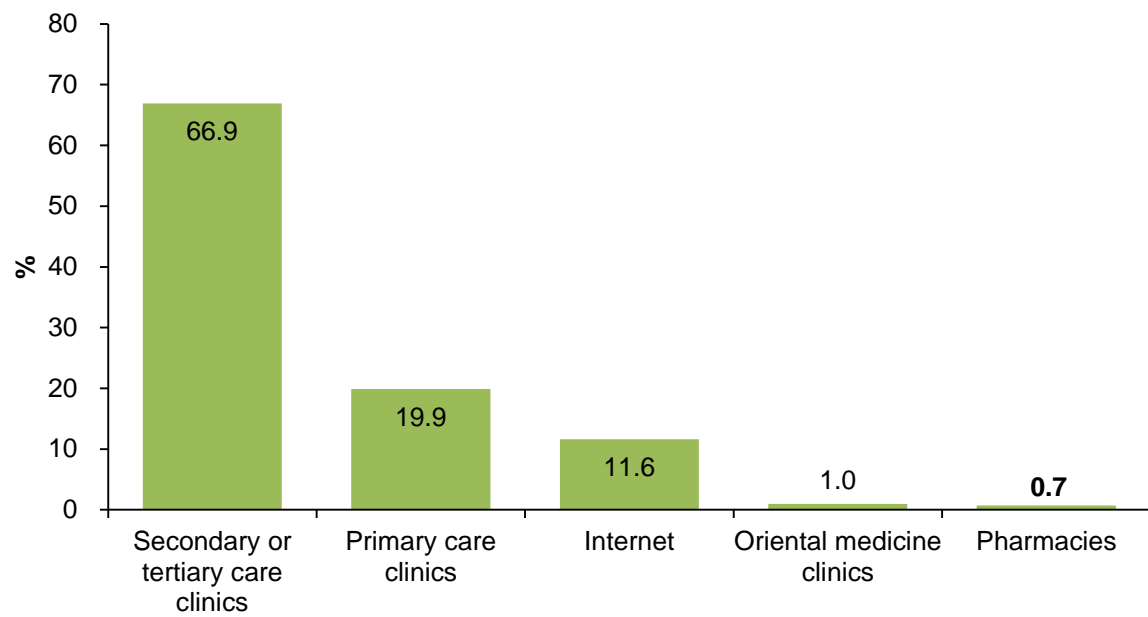

Supplement: S8 Fig — (PDF) [file pone.0222264.s008.pdf]
