# Supplementary material for: Heart failure awareness in the Korean general population: Results from the nationwide survey
Source: PLoS One. 2019 Sep 6;14(9):e0222264. doi: 10.1371/journal.pone.0222264 (PMC6731018; doi:10.1371/journal.pone.0222264)

S3 Fig. Response to the question, ‘how soon will you go to the hospital if you experience breathlessness, tiredness, or swollen ankles?’

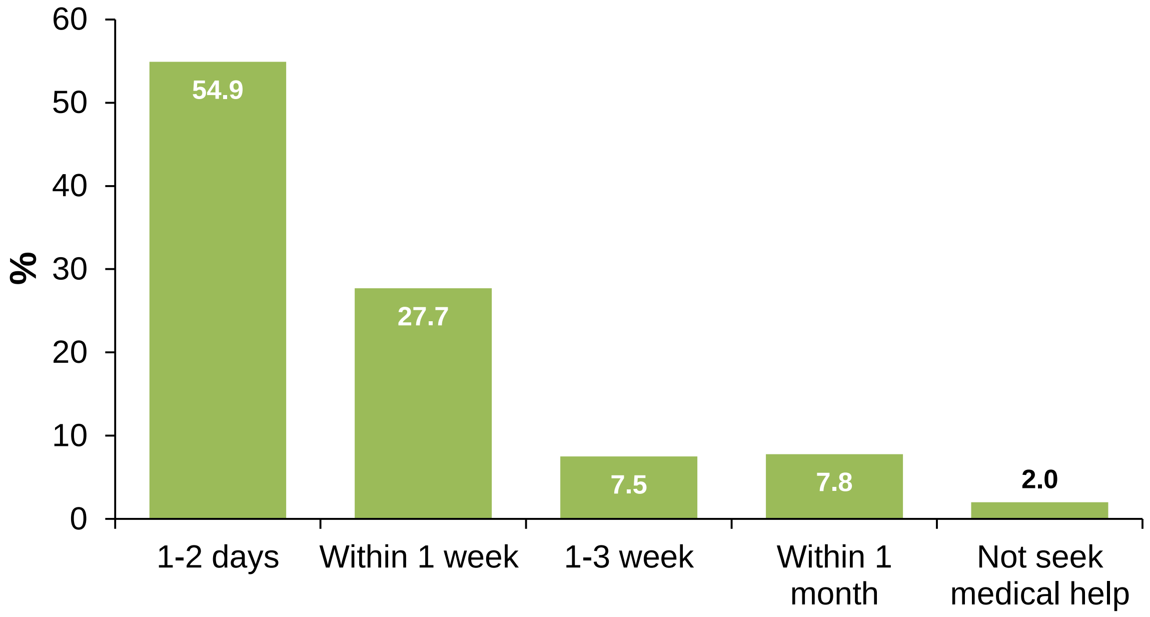

Supplement: S3 Fig — (PDF) [file pone.0222264.s003.pdf]
