# Supplementary material for: Heart failure awareness in the Korean general population: Results from the nationwide survey
Source: PLoS One. 2019 Sep 6;14(9):e0222264. doi: 10.1371/journal.pone.0222264 (PMC6731018; doi:10.1371/journal.pone.0222264)

S6 Fig. Response to the question, 'most likely disease that has the highest mortality within 5 years after the diagnosis'

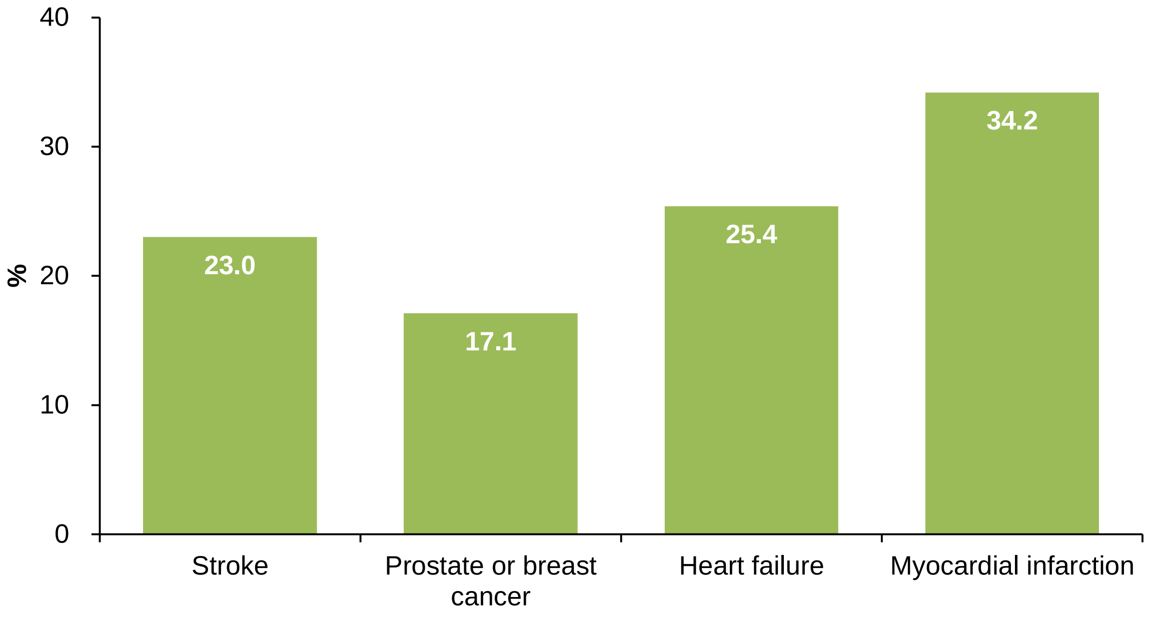

Supplement: S6 Fig — (PDF) [file pone.0222264.s006.pdf]
