# Supplementary material for: Heart failure awareness in the Korean general population: Results from the nationwide survey
Source: PLoS One. 2019 Sep 6;14(9):e0222264. doi: 10.1371/journal.pone.0222264 (PMC6731018; doi:10.1371/journal.pone.0222264)
Supplement: S1 Table — (PDF) [file pone.0222264.s009.pdf]

S1 Table. Questionnaire items for identifying eligibility of the study population

Items	Questions	Answers
**Eligibility of the study participants**		
E-Q1	Sex	1. Men<br>2. Women
E-Q2	Age	__ years
E-Q3	Place of residence*	1. Seoul<br>2. Busan<br>3. Daegu<br>4. Incheon<br>5. Gwangju<br>6. Daejeon<br>7. Ulsan<br>8. Gyeonggi<br>9. Gangwon<br>10. Chungbuk<br>11. Chungnam<br>12. Jeonbuk<br>13. Jeonnam<br>14. Gyeongbuk<br>15. Gyeongnam<br>16. Jeju
E-Q4	Urbanization level of residence	1. Urban (*dong*)<br>2. Rural (*eup, myeon, ri*)

*Residence further divided into 2 categories as metropolitan cities (Seoul, Busan, Daegu, Incheon, Gwangju, Daejeon, and Ulsan) and provinces (Gyeonggi, Gangwon, Chungbuk, Chungnam, Jeonbuk, Jeonnam, Gyeongbuk, Gyenognam, and Jeju).
