# Supplementary material for: Heart failure awareness in the Korean general population: Results from the nationwide survey
Source: PLoS One. 2019 Sep 6;14(9):e0222264. doi: 10.1371/journal.pone.0222264 (PMC6731018; doi:10.1371/journal.pone.0222264)
Supplement: S2 Table — (PDF) [file pone.0222264.s010.pdf]

S2 Table. Main questionnaire contents

Items	Questions	Answers
**Before the definition of heart failure is given**		
Q1	What disease do you think of if someone has the following symptoms?<br>Symptom: chest heaviness and tightness that occur during exertion and disappears with rest	1. Angina or myocardial infarction<br>2. Gastro-intestinal disorder<br>3. Other heart diseases<br>4. Lung disorders<br>5. I do not know
Q2	What disease do you think of if someone has the following symptoms?<br>Symptom: facial paralysis, double vision, and/or sudden unilateral weakness in the arm	1. Stroke<br>2. Parkinson, epilepsy, other brain disease<br>3. Other heart diseases<br>4. Angina or myocardial infarction<br>5. I do not know
Q3	What disease do you think of if someone has the following symptoms?<br>Symptom: breathlessness with low level activity, tiredness, and swollen ankles	1. Heart failure<br>2. Heart in general<br>3. Angina or myocardial infarction<br>4. Lung disorders<br>5. I do not know
Q4	Have you ever heard of heart failure?	1. Yes<br>2. No
Q5	What do you think is the best representation of heart failure?	1. Heart having a blood- and oxygen-deprived state due to a clot formed in the vessel<br>2. Heart rhythm abnormality<br>3. Weakness of the heart due to the natural course of aging<br>4. Heart cannot pump enough blood

		around the body<br>5. I don't know
Q6	What do you think the severity is if you have the following symptoms: breathlessness, tiredness, or swollen ankles?	1. Serious illness<br>2. Slightly serious illness<br>3. Minor illness<br>4. I do not know
Q7	How soon will you go to the hospital if you feel breathlessness, tiredness, or swollen ankles?	1. 1-2 days<br>2. within 1 week<br>3. 1-3 weeks<br>4. 1 month<br>5. Never (I will not go to the hospital)
Q8	Is there anyone among you or your family members who are suffering from heart disease?	1. Yes (→ to Q8-1)<br>2. No (→ to Q9)
Q8-1	What is his/her diagnosis?	1. Angina or myocardial infarction<br>2. Heart failure<br>3. Arrhythmia (abnormality of heart rhythm)<br>4. Valvular heart disease<br>5. I do not know

Definition of heart failure is given.

Heart failure is a state when the heart is unable to sufficiently pump to maintain the blood flow to meet the body's needs. Typical symptoms/signs include shortness of breath, excessive tiredness, and ankle swelling.

After the announcement of the heart failure definition

Q9	Do you agree that heart failure is a normal aging process?	1. Yes<br>2. No
Q10	Which of the following conditions is a precipitating cause for developing heart failure?	1. Hypertension<br>2. Lung disorder

Q11	Which of the following do you think is the correct life time risk of developing heart failure?	1. 1 in 100 people<br>2. 5 in 100 people<br>3. 10 in 100 people<br>4. 20 in 100 people
Q12	Which of the following diseases is most likely to have the highest mortality within 5 years after diagnosis?	1. Stroke<br>2. Prostate cancer or breast cancer<br>3. Heart failure<br>4. Myocardial infarction
Q13	Suppose one of your friends, colleagues, or neighbors suffers from heart failure. Are you worried that they might suddenly die?	1. Yes<br>2. No<br>3. I do not know
Q14	Acute heart failure is a sudden worsening of the signs and symptoms of heart failure. Which of the following is correct for the post-discharge 1-year mortality from acute heart failure?	1. 2 in 100 people might die<br>2. 5 in 100 people might die<br>3. 10 in 100 people might die<br>4. 20 in 100 people might die<br>5. I do not know
Q15	What is the readmission rate within 1 year after discharge from heart failure?	1. 2 in 100 people<br>2. 5 in 100 people<br>3. 10 in 100 people<br>4. 20 in 100 people<br>5. I do not know
Q16	What do you think the average healthcare costs per admission are from acute heart failure?<br>(medical bills that include both out-of-pocket and insurance copayments)	1. 1,000,000 Korean won (KRW)<br>2. 2,000,000 KRW<br>3. 3,000,000 KRW<br>4. 4,000,000 KRW<br>5. ≥5,000,000 KRW
Q17	What disease do you think will have the greatest impact on the quality of life? Please select only one.	1. Diabetes<br>2. Arthritis<br>3. Heart failure

		4. Hypertension
Q18	If you were a heart failure patient, which of following treatments would you prefer?	1. Treatment that could improve the quality of life<br>2. Treatment that allows you to live longer<br>3. I cannot decide
Q19	Do you agree that 'current heart failure medications could reduce death from heart failure'?	1. Yes<br>2. No<br>3. I do not know
Q20	Do you agree that 'current heart failure medications could improve the quality of life in patients with heart failure'?	1. Yes<br>2. No<br>3. I do not know
Q21	Do you agree that 'current heart failure medications could prevent the occurrence of heart failure'?	1. Yes<br>2. No<br>3. I do not know
Q22	Suppose one of your friends, colleagues, or neighbors suffers from heart failure. Do you think that they should live quietly and reduce all physical activity?	1. Yes<br>2. No<br>3. I do not know
Q23	If you need information about heart failure, where would you visit?	1. Primary care clinic<br>2. Secondary or tertiary care clinic<br>3. Oriental medicine clinic<br>4. Pharmacy<br>5. Internet
