# Supplementary material for: Heart failure awareness in the Korean general population: Results from the nationwide survey
Source: PLoS One. 2019 Sep 6;14(9):e0222264. doi: 10.1371/journal.pone.0222264 (PMC6731018; doi:10.1371/journal.pone.0222264)
Supplement: S3 Table — (PDF) [file pone.0222264.s011.pdf]

S3 Table. Questionnaire items for identifying demographic characteristics

Items	Questions	Answers
D-Q1	Educational attainment	1. Middle school or less<br>2. High school<br>3. College or more
D-Q2	Household income (HI)	1. HI ≤ 1,000,000 KRW<br>2. 1,000,000 < HI ≤ 2,000,000 KRW<br>3. 2,000,000 < HI ≤ 3,000,000 KRW<br>4. 3,000,000 < HI ≤ 4,000,000 KRW<br>5. 4,000,000 < HI ≤ 5,000,000 KRW<br>6. HI > 5,000,000 KRW
D-Q3	Comorbidity of the responder	1. Hypertension<br>2. Diabetes<br>3. Dyslipidemia<br>4. None
D-Q4	Presence of heart failure of the responder or family member (parents, siblings, children)	1. Responder<br>2. Responder’s family member<br>3. None
D-Q5	Smoking	1. Never<br>2. Ex-smoker (quit more than 1 year)<br>3. Ex-smoker (quit within 1 year)<br>4. Current smoker: average __ pack years
D-Q6	Alcohol consumption (during last year)	1. Yes (→ to Q6-1)<br>2. None (→ end of questionnaire)
D-Q6-1	Frequency of alcohol consumption	1. Less than once a month<br>2. About once a month

	3. About 2-4 times a month<br>4. About 2-3 times a week<br>5. 4 times and more a week
