# Supplementary material for: Heart failure awareness in the Korean general population: Results from the nationwide survey
Source: PLoS One. 2019 Sep 6;14(9):e0222264. doi: 10.1371/journal.pone.0222264 (PMC6731018; doi:10.1371/journal.pone.0222264)
Supplement: S4 Table — (PDF) [file pone.0222264.s012.pdf]

**S4 Table. Clinical and demographic characteristics of the study population (N = 1,032)**

| | Number | Percent |
|---|---|---|
| Sex | | |
| Male | 522 | 50.6 |
| Female | 510 | 49.4 |
| Age (binary) | | |
| 30-64, years | 535 | 51.8 |
| ≥ 65, years | 497 | 48.2 |
| Age (decades) | | |
| 30-39, years | 157 | 15.2 |
| 40-49, years | 146 | 14.1 |
| 50-59, years | 161 | 15.6 |
| 60-69, years | 341 | 33.0 |
| 70-79, years | 175 | 17.0 |
| ≥ 80, years | 52 | 5.0 |
| Place of residence[*] | | |
| Metropolitan cities | 517 | 50.1 |
| Provinces | 515 | 49.9 |
| Urbanization level of residence | | |
| Urban (*dong*) | 887 | 85.9 |
| Rural (*eup, myeon, ri*) | 145 | 14.1 |
| Educational attainment | | |
| Middle school or less | 207 | 20.1 |
| High school | 309 | 29.9 |
| College or more | 504 | 48.8 |
| Do not want to answer | 12 | 1.2 |
| Household income (HI, KRW 1,000[†]) | | |
| HI ≤ 1,000 | 87 | 8.4 |
| 1,000 < HI ≤ 2,000 | 111 | 10.8 |

| | | |
|---|---|---|
| 2,000 < HI ≤ 3,000 | 248 | 24.0 |
| 3,000 < HI ≤ 4,000 | 229 | 22.2 |
| 4,000 < HI ≤ 5,000 | 156 | 15.1 |
| HI > 5,000 | 164 | 15.9 |
| Do not want to answer | 37 | 3.6 |
| Presence of comorbidity[‡] | | |
| Yes | 356 | 34.5 |
| No | 676 | 65.5 |
| Presence of heart failure[§] | | |
| Yes | 41 | 4.0 |
| No | 991 | 96.0 |

Data are expressed as numbers with percentages. [*]Residences are devided into 2 categories: metropolitan cities (Seoul, Busan, Daegu, Incheon, Gwangju, Daejeon, and Ulsan) and provinces (Gyeonggi, Gangwon, Chungbuk, Chungnam, Jeonbuk, Jeonnam, Gyeongbuk, Gyeongnam, and Jeju). [†]US $1=1113.5 Korean won (KRW), October 2018. [‡]Comorbidities (of hypertension, diabetes, and dyslipidemia) of respondents were surveyed. In detail, 236 (22.9%) had hypertension, 63 (6.1%) had diabetes, and 57 (5.5%) had dyslipidemia. [§]The presence of heart failure either respondernts or family member was surveyed.
