# Supplementary material for: Heart failure awareness in the Korean general population: Results from the nationwide survey
Source: PLoS One. 2019 Sep 6;14(9):e0222264. doi: 10.1371/journal.pone.0222264 (PMC6731018; doi:10.1371/journal.pone.0222264)
Supplement: S5 Table — (PDF) [file pone.0222264.s013.pdf]

S5 Table. Differences in the awareness of heart failure symptoms among subgroups (Q4)

Q4: Have you ever heard of heart failure?			
Answer	Yes	No	p-value
Total (n = 1,032)	827 (80.1)	205 (19.9)	-
Sex			0.001
Male	440 (84.3)	82 (15.7)	
Female	387 (75.9)	123 (24.1)	
Age (binary)			0.001
30-64 years	450 (84.1)	85 (15.9)	
≥ 65 years	377 (75.9)	120 (24.1)	
Age (decades)			<0.001
30-39 years	124 (79.0)	33 (21.0)	
40-49 years	132 (90.4)	14 (9.6)	
50-59 years	137 (85.1)	24 (14.9)	
60-69 years	282 (82.7)	59 (17.3)	
70-79 years	125 (71.4)	50 (28.6)	
≥ 80 years	27 (51.9)	25 (48.1)	
Urbanization level of residence			0.014
Urban (*dong*)	722 (81.4)	165 (18.6)	
Rural (*eup, myeon, ri*)	84 (72.4)	40 (27.6)	
Educational attainment			<0.001
Middle school or less	134 (64.7)	73 (35.3)	
High school	248 (80.3)	61 (19.7)	
College or more	436 (86.5)	68 (13.5)	
Do not want to say	9 (75.0)	3 (25.0)	
Household income (HI, KRW 1,000*)			<0.001
HI ≤ 1,000	52 (59.8)	35 (40.2)	
1,000 < HI ≤ 2,000	85 (76.6)	26 (23.4)	
2,000 < HI ≤ 3,000	195 (78.6)	53 (21.4)	

3,000 < HI ≤ 4,000	190 (83.0)	39 (17.0)	
4,000 < HI ≤ 5,000	138 (88.5)	18 (11.5)	
HI > 5,000	139 (84.8)	25 (15.2)	
Do not want to answer	28 (75,7)	9 (24.3)	
Presence of comorbidity†			ns
Yes	274 (77.0)	82 (23.0)	
No	553 (81.8)	123 (18.2)	

Data were the number (percentage). *US $1=1113.5 Korean won (KRW), October 2018. †Comorbidities (any of hypertension, diabetes, dyslipidemia) of the responders were surveyed. ns = non-significant.
