# Supplementary material for: Heart failure awareness in the Korean general population: Results from the nationwide survey
Source: PLoS One. 2019 Sep 6;14(9):e0222264. doi: 10.1371/journal.pone.0222264 (PMC6731018; doi:10.1371/journal.pone.0222264)
Supplement: S6 Table — (PDF) [file pone.0222264.s014.pdf]

## S6 Table. Differences in the awareness of heart failure symptoms among subgroups (Q5)

| Q5: What do you think is the best presentation for failure? | | | | | | |
|---|---|---|---|---|---|---|
| | Answer | | | | | |
| | Heart having a blood- and oxygen-deprived state due to a clot formed in the vessel | Heart rhythm abnormality | Weakness of the heart due to the natural course of aging | Heart cannot pump enough blood around the body (correct answer) | I do not know | p-value |
| Data are presented with % | 21.7 | 11.8 | 5.7 | 47.3 | 13.5 | - |
| Sex | | | | | | ns |
| Male | 23.4 | 10.9 | 6.5 | 48.5 | 10.7 | |
| Female | 20.0 | 12.7 | 4.9 | 46.1 | 16.3 | |
| Age (binary) | | | | | | < 0.001 |
| 30-64 years | 23.7 | 12.5 | 5.4 | 50.1 | 8.2 | |
| ≥ 65 years | 19.5 | 11.1 | 6.0 | 44.3 | 19.1 | |
| Urbanization level of residence | | | | | | < 0.001 |
| Urban (*dong*) | 23.0 | 11.8 | 5.9 | 48.1 | 11.2 | |
| Rural (*eup, myeon, ri*) | 13.8 | 11.7 | 4.8 | 42.1 | 27.6 | |
| Educational attainment | | | | | | < 0.001 |
| Middle school or less | 15.0 | 13.5 | 4.3 | 37.2 | 30.0 | |
| High school | 24.9 | 12.9 | 7.1 | 43.7 | 11.3 | |
| College or more | 22.4 | 10.5 | 5.6 | 53.6 | 7.9 | |
| Do not want to say | 25.0 | 8.3 | 0.0 | 50.0 | 16.7 | |
| Household income (HI, KRW 1,000[*]) | | | | | | < 0.001 |
| HI ≤ 1,000 | 13.8 | 4.6 | 2.3 | 36.8 | 42.5 | |
| 1,000 < HI ≤ 2,000 | 27.0 | 12.6 | 4.5 | 43.2 | 12.6 | |
| 2,000 < HI ≤ 3,000 | 22.2 | 14.9 | 7.7 | 41.5 | 13.7 | |
| 3,000 < HI ≤ 4,000 | 22.3 | 14.0 | 4.8 | 47.2 | 11.8 | |
| 4,000 < HI ≤ 5,000 | 23.1 | 10.3 | 5.8 | 55.8 | 5.1 | |
| HI > 5,000 | 21.3 | 8.5 | 6.7 | 55.5 | 7.9 | |
| Do not want to say | 13.5 | 13.5 | 5.4 | 51.4 | 16.2 | |
| Presence of comorbidity[†] | | | | | | < 0.05 |
| Yes | 19.9 | 10.7 | 8.1 | 44.4 | 16.9 | |
| No | 22.6 | 12.4 | 4.4 | 48.8 | 11.7 | |

[*]US $1=1113.5 Korean won (KRW), October 2018. [†]Comorbidities (any of hypertension, diabetes, dyslipidemia) of the responders were

surveyed.

ns = non-significant.
