# Supplementary material for: Heart failure awareness in the Korean general population: Results from the nationwide survey
Source: PLoS One. 2019 Sep 6;14(9):e0222264. doi: 10.1371/journal.pone.0222264 (PMC6731018; doi:10.1371/journal.pone.0222264)
Supplement: S7 Table — (PDF) [file pone.0222264.s015.pdf]

S7 Table. Differences in the awareness of heart failure symptoms among subgroups (Q6)

Q6: What do you think the severity is if you have the following symptoms: breathlessness, tiredness, or swollen ankles?					
	Answer				
	Serious illness	Slightly serious illness	Minor illness	I do not know	p-value
Data are presented with %	62.0	27.3	6.2	4.5	-
Sex					< 0.05
Male	66.1	25.7	5.0	3.3	
Female	57.8	29.0	7.5	5.7	
Age (binary)					< 0.01
30-64 years	62.8	28.6	6.5	2.1	
≥ 65 years	61.2	26.0	5.8	7.0	
Urbanization level of residence					< 0.001
Urban (*dong*)	60.8	28.9	6.8	3.6	
Rural (*eup, myeon, ri*)	69.7	17.9	2.8	9.7	
Educational attainment					< 0.001
Middle school or less	60.4	22.7	4.3	12.6	
High school	56.6	32.0	7.1	4.2	
College or more	65.7	26.6	6.5	1.2	
Do not want to say	75.0	16.7	0.0	8.3	
Household income (HI, KRW 1,000*)					< 0.001
HI ≤ 1,000	63.2	17.2	1.1	18.4	
1,000 < HI ≤ 2,000	59.5	31.5	5.4	3.6	
2,000 < HI ≤ 3,000	54.4	30.6	9.7	5.2	
3,000 < HI ≤ 4,000	61.6	29.7	7.0	1.7	
4,000 < HI ≤ 5,000	63.5	28.2	5.8	2.6	
HI > 5,000	70.7	22.0	4.9	2.4	
Do not want to say	75.7	21.6	0.0	2.7	
Presence of comorbidity†					< 0.05
Yes	66.6	22.2	5.6	5.6	
No	59.6	30.0	6.5	3.8	

*US $1=1113.5 Korean won (KRW), October 2018. †Comorbidities (any of hypertension, diabetes, dyslipidemia) of the responders were surveyed.

ns = non-significant.
