# Supplementary material for: Heart failure awareness in the Korean general population: Results from the nationwide survey
Source: PLoS One. 2019 Sep 6;14(9):e0222264. doi: 10.1371/journal.pone.0222264 (PMC6731018; doi:10.1371/journal.pone.0222264)
Supplement: S8 Table — (PDF) [file pone.0222264.s016.pdf]

S8 Table. Differences in the awareness of heart failure symptoms among subgroups (Q7)

Q7: How soon will you go to the hospital if you feel breathlessness, tiredness, or swollen ankles?						
	Answer					
	1-2 days	within 1 week	1-3 weeks	1 month	Never (I will not go to the hospital)	p-value
Data are presented with %	54.9	27.7	7.5	7.8	2.0	-
Sex						< 0.05
Male	59.2	25.5	7.1	5.9	2.3	
Female	50.6	30.0	7.8	9.8	1.8	
Age (binary)						ns
30-64 years	51.8	28.6	8.4	9.0	2.2	
≥ 65 years	58.4	26.8	6.4	6.6	1.8	
Urbanization level of residence						< 0.001
Urban (*dong*)	53.3	28.7	8.2	7.6	2.1	
Rural (*eup, myeon, ri*)	64.8	21.4	2.8	9.7	1.4	
Educational attainment						ns
Middle school or less	60.4	25.6	5.3	7.7	1.0	
High school	50.8	28.2	11.0	7.8	2.3	
College or more	54.8	28.4	6.3	8.1	2.4	
Do not want to say	75.0	25.0	0.0	0.0	0.0	
Household income (HI, KRW 1,000[*])						< 0.01
HI ≤ 1,000	70.1	16.1	3.4	8.0	2.3	
1,000 < HI ≤ 2,000	52.3	28.8	5.4	12.6	0.9	
2,000 < HI ≤ 3,000	45.6	35.5	10.5	5.6	2.8	
3,000 < HI ≤ 4,000	52.8	31.4	8.7	5.2	1.7	
4,000 < HI ≤ 5,000	55.8	23.1	10.9	8.3	1.9	
HI > 5,000	61.0	23.2	3.0	10.4	2.4	
Do not want to say	73.0	16.2	0.0	10.8	0.0	
Presence of comorbidity[†]						< 0.05
Yes	61.0	22.8	8.1	6.2	2.0	
No	51.8	30.3	7.1	8.7	2.1	

[*]US $1=1113.5 Korean won (KRW), October 2018. [†]Comorbidities (any of hypertension, diabetes, dyslipidemia) of the responders were surveyed.

ns = non-significant.
