# Supplementary material for: Heart failure awareness in the Korean general population: Results from the nationwide survey
Source: PLoS One. 2019 Sep 6;14(9):e0222264. doi: 10.1371/journal.pone.0222264 (PMC6731018; doi:10.1371/journal.pone.0222264)
Supplement: S9 Table — (PDF) [file pone.0222264.s017.pdf]

S9 Table. Differences in the awareness of heart failure symptoms among subgroups (Q9)

Q9: Do you agree that heart failure is a normal aging process?			
Answer	Yes	No	p-value
Data are presented with %	35.3	64.7	-
Sex			ns
Male	34.5	65.5	
Female	36.1	63.9	
Age (binary)			< 0.05
30-64 years	31.6	68.4	
≥ 65 years	39.2	60.8	
Age (decades)			< 0.05
30-39 years	29.3	70.7	
40-49 years	27.4	72.6	
50-59 years	34.2	65.8	
60-69 years	36.1	63.9	
70-79 years	45.1	54.9	
≥ 80 years	40.4	59.6	
Urbanization level of residence			ns
Urban (*dong*)	34.9	65.1	
Rural (*eup*, *myeon*, *ri*)	37.2	62.8	
Educational attainment			< 0.01
Middle school or less	43.0	57.0	
High school	38.5	61.5	
College or more	29.8	70.2	
Do not want to say	50.0	50.0	
Household income (HI, KRW 1,000*)			ns
HI ≤ 1,000	42.5	57.5	
1,000 < HI ≤ 2,000	36.0	64.0	
2,000 < HI ≤ 3,000	37.1	62.9	
3,000 < HI ≤ 4,000	32.3	67.7	
4,000 < HI ≤ 5,000	32.7	67.3	
HI > 5,000	34.8	65.2	
Do not want to say	35.1	64.9	
Presence of comorbidity†			ns
Yes	36.5	63.5	
No	34.6	65.4	

*US $1=1113.5 Korean won (KRW), October 2018. †Comorbidities (any of hypertension, diabetes, dyslipidemia) of the responders were

surveyed.

ns = non-significant.
