# Supplementary material for: Heart failure awareness in the Korean general population: Results from the nationwide survey
Source: PLoS One. 2019 Sep 6;14(9):e0222264. doi: 10.1371/journal.pone.0222264 (PMC6731018; doi:10.1371/journal.pone.0222264)
Supplement: S10 Table — (PDF) [file pone.0222264.s018.pdf]

S10 Table. Differences in the awareness of heart failure symptoms among subgroups (Q10)

Q10: Which of the following conditions is a precipitating cause for developing heart failure?				
Answer	Hypertension	Lung disorder	Do not know	p-value
Data are presented with %	74.1	15.4	10.5	-
Sex				< 0.05
Male	77.2	14.6	8.2	
Female	71.0	16.3	12.7	
Age (binary)				< 0.001
30-64 years	82.4	11.6	6.0	
≥ 65 years	65.2	19.5	15.3	
Age (decades)				< 0.001
30-39 years	87.9	8.9	3.2	
40-49 years	83.6	11.6	4.8	
50-59 years	82.0	10.6	7.5	
60-69 years	68.0	20.5	11.4	
70-79 years	65.7	20.6	13.7	
≥ 80 years	50.0	9.6	40.4	
Urbanization level of residence				ns
Urban (*dong*)	74.4	15.8	9.8	
Rural (*eup, myeon, ri*)	72.4	13.1	14.5	
Educational attainment				< 0.001
Middle school or less	58.0	19.3	22.7	
High school	68.0	20.4	11.7	
College or more	84.3	10.9	4.8	
Do not want to say	83.3	8.3	8.3	
Household income (HI, KRW 1,000[*])				< 0.001
HI ≤ 1,000	52.9	16.1	31.0	
1,000 < HI ≤ 2,000	74.8	17.1	8.1	
2,000 < HI ≤ 3,000	66.9	21.0	12.1	
3,000 < HI ≤ 4,000	79.0	14.4	6.6	
4,000 < HI ≤ 5,000	77.6	13.5	9.0	
HI > 5,000	83.5	10.4	6.1	
Do not want to say	83.8	8.1	8.1	
Presence of comorbidity[†]				< 0.01
Yes	69.4	17.7	12.9	
No	76.6	14.2	9.2	

[*]US $1=1113.5 Korean won (KRW), October 2018. [†]Comorbidities (any of hypertension, diabetes, dyslipidemia) of the responders were

surveyed.

ns = non-significant.
