# Supplementary material for: Heart failure awareness in the Korean general population: Results from the nationwide survey
Source: PLoS One. 2019 Sep 6;14(9):e0222264. doi: 10.1371/journal.pone.0222264 (PMC6731018; doi:10.1371/journal.pone.0222264)
Supplement: S11 Table — (PDF) [file pone.0222264.s019.pdf]

S11 Table. Differences in the awareness of heart failure symptoms among subgroups (Q11)

Q11: Which of the following do you think is the correct life time risk of developing heart failure?						
	Answer					
	1 in 100 people	5 in 100 people	10 in 100 people	20 in 100 people (correct)	Do not know	p-value
Data are presented with %	11.8	31.2	32.5	21.4	3.1	-
Sex						ns
Male	10.5	32.0	32.4	21.8	3.3	
Female	13.1	30.4	32.5	21.0	2.9	
Age (binary)						< 0.01
30-64 years	10.8	31.8	31.6	24.5	1.3	
≥ 65 years	12.9	30.6	33.4	18.1	5.0	
Age (decades)						< 0.001
30-39 years	8.9	26.8	33.8	29.3	1.3	
40-49 years	10.3	36.3	30.8	21.9	0.7	
50-59 years	12.4	32.9	29.2	24.2	1.2	
60-69 years	12.6	33.1	33.1	17.6	3.5	
70-79 years	12.0	29.7	34.9	19.4	4.0	
≥ 80 years	17.3	17.3	30.8	19.2	15.4	
Urbanization level of residence						< 0.001
Urban (*dong*)	12.9	32.5	32.2	20.5	1.9	
Rural (*eup, myeon, ri*)	5.5	23.4	33.8	26.9	10.3	
Educational attainment						< 0.001
Middle school or less	17.4	28.5	29.0	18.8	6.3	
High school	14.9	35.6	29.8	17.8	1.9	
College or more	7.7	29.4	35.3	25.0	2.6	
Do not want to say	8.3	41.7	41.7	8.3	0.0	
Household income (HI, KRW 1,000*)						< 0.001
HI ≤ 1,000	11.5	19.5	35.6	23.0	10.3	
1,000 < HI ≤ 2,000	7.2	31.5	40.5	18.9	1.8	
2,000 < HI ≤ 3,000	15.7	32.7	29.0	19.8	2.8	
3,000 < HI ≤ 4,000	12.7	32.3	33.6	20.1	1.3	
4,000 < HI ≤ 5,000	14.1	31.4	29.5	23.7	1.3	
HI > 5,000	6.1	35.4	32.3	23.8	2.4	
Do not want to say	10.8	21.6	29.7	24.3	13.5	

Presence of comorbidity[†]						ns
Yes	13.2	29.5	31.2	21.9	4.2	
No	11.1	32.1	33.1	21.2	2.5	

[*]US $1=1113.5 Korean won (KRW), October 2018. [†]Comorbidities (any of hypertension, diabetes, dyslipidemia) of the responders were surveyed.

ns = non-significant.
