# Supplementary material for: Heart failure awareness in the Korean general population: Results from the nationwide survey
Source: PLoS One. 2019 Sep 6;14(9):e0222264. doi: 10.1371/journal.pone.0222264 (PMC6731018; doi:10.1371/journal.pone.0222264)
Supplement: S12 Table — (PDF) [file pone.0222264.s020.pdf]

## S12 Table. Differences in the awareness of heart failure symptoms among subgroups (Q12)

| Q12: Which of the following diseases is most likely to have the highest mortality within 5 years after diagnosis? | | | | | | |
|---|---|---|---|---|---|---|
| | Answer | | | | | |
| | Stroke | Prostate or breast cancer | Heart failure (correct) | Myocardial infarction | Do not know | p-value |
| Data are presented with % | 23.0 | 17.1 | 25.4 | 34.2 | 0.4 | - |
| Sex | | | | | | ns |
| Male | 21.5 | 17.0 | 26.1 | 34.9 | 0.6 | |
| Female | 24.5 | 17.1 | 24.7 | 33.5 | 0.2 | |
| Age (binary) | | | | | | < 0.05 |
| 30-64 years | 19.8 | 15.7 | 26.9 | 37.2 | 0.4 | |
| ≥ 65 years | 26.4 | 18.5 | 23.7 | 31.0 | 0.4 | |
| Age (decades) | | | | | | < 0.05 |
| 30-39 years | 17.8 | 8.3 | 30.6 | 43.3 | 0.0 | |
| 40-49 years | 21.9 | 15.8 | 28.1 | 33.6 | 0.7 | |
| 50-59 years | 18.6 | 18.0 | 24.8 | 38.5 | 0.0 | |
| 60-69 years | 24.0 | 18.8 | 23.8 | 32.8 | 0.6 | |
| 70-79 years | 28.6 | 19.4 | 24.6 | 27.4 | 0.0 | |
| ≥ 80 years | 28.8 | 25.0 | 17.3 | 26.9 | 1.9 | |
| Urbanization level of residence | | | | | | < 0.05 |
| Urban (*dong*) | 22.8 | 18.2 | 24.9 | 33.9 | 0.2 | |
| Rural (*eup*, *myeon*, *ri*) | 24.1 | 10.3 | 28.3 | 35.9 | 1.4 | |
| Educational attainment | | | | | | < 0.001 |
| Middle school or less | 27.1 | 23.7 | 26.1 | 22.7 | 0.5 | |
| High school | 21.7 | 24.3 | 20.4 | 33.7 | 0.0 | |
| College or more | 21.6 | 9.7 | 28.6 | 39.5 | 0.6 | |
| Do not want to say | 41.7 | 25.0 | 8.3 | 25.0 | 0.0 | |
| Household income (HI, KRW 1,000[*]) | | | | | | < 0.05 |
| HI ≤ 1,000 | 28.7 | 17.2 | 21.8 | 31.0 | 1.1 | |
| 1,000 < HI ≤ 2,000 | 20.7 | 13.5 | 30.6 | 35.1 | 0.0 | |
| 2,000 < HI ≤ 3,000 | 24.2 | 21.4 | 23.0 | 31.5 | 0.0 | |
| 3,000 < HI ≤ 4,000 | 21.0 | 16.2 | 26.6 | 35.8 | 0.4 | |
| 4,000 < HI ≤ 5,000 | 20.5 | 23.7 | 24.4 | 31.4 | 0.0 | |
| HI > 5,000 | 21.3 | 9.1 | 25.0 | 43.9 | 0.6 | |
| Do not want to say | 37.8 | 10.8 | 32.4 | 16.2 | 2.7 | |
| Presence of comorbidity[†] | | | | | | ns |

| | | | | | |
|---|---|---|---|---|---|
| Yes | 23.3 | 17.7 | 25.6 | 33.1 | 0.3 |
| No | 22.8 | 16.7 | 25.3 | 34.8 | 0.4 |

[*]US $1=1113.5 Korean won (KRW), October 2018. [†]Comorbidities (any of hypertension, diabetes, dyslipidemia) of the responders were surveyed.

ns = non-significant.
