# Supplementary material for: Heart failure awareness in the Korean general population: Results from the nationwide survey
Source: PLoS One. 2019 Sep 6;14(9):e0222264. doi: 10.1371/journal.pone.0222264 (PMC6731018; doi:10.1371/journal.pone.0222264)
Supplement: S13 Table — (PDF) [file pone.0222264.s021.pdf]

S13 Table. Differences in the awareness of heart failure symptoms among subgroups (Q13)

Q13: Suppose one of your friends, colleagues, or neighbors suffers from heart failure. Are you worried that they might suddenly die?				
Answer	Yes	No	Do not know	p-value
Data are presented with %	62.4	23.6	14.0	-
Sex				ns
Male	64.4	23.2	12.5	
Female	60.4	24.1	15.5	
Age (binary)				< 0.05
30-64 years	64.5	24.7	10.8	
≥ 65 years	60.2	22.5	17.3	
Age (decades)				< 0.001
30-39 years	70.7	24.2	5.1	
40-49 years	64.4	25.3	10.3	
50-59 years	61.5	24.2	14.3	
60-69 years	63.3	24.0	12.6	
70-79 years	55.4	23.4	21.1	
≥ 80 years	51.9	13.5	34.6	
Urbanization level of residence				< 0.001
Urban (*dong*)	61.8	25.5	12.7	
Rural (*eup*, *myeon*, *ri*)	66.2	12.4	21.4	
Educational attainment				< 0.001
Middle school or less	51.7	21.7	26.6	
High school	59.2	25.9	14.9	
College or more	68.8	23.2	7.9	
Do not want to say	58.3	16.7	25.0	
Household income (HI, KRW 1,000[*])				< 0.001
HI ≤ 1,000	51.7	12.6	35.6	
1,000 < HI ≤ 2,000	69.4	20.7	9.9	
2,000 < HI ≤ 3,000	57.3	33.5	9.3	
3,000 < HI ≤ 4,000	59.4	25.3	15.3	
4,000 < HI ≤ 5,000	63.5	22.4	14.1	
HI > 5,000	75.0	18.3	6.7	
Do not want to say	59.5	10.8	29.7	
Presence of comorbidity[†]				ns
Yes	63.2	20.8	16.0	

No	62.0	25.1	12.9

*US $1=1113.5 Korean won (KRW), October 2018. †Comorbidities (any of hypertension, diabetes, dyslipidemia) of the responders were surveyed.

ns = non-significant.
