# Supplementary material for: Heart failure awareness in the Korean general population: Results from the nationwide survey
Source: PLoS One. 2019 Sep 6;14(9):e0222264. doi: 10.1371/journal.pone.0222264 (PMC6731018; doi:10.1371/journal.pone.0222264)
Supplement: S14 Table — (PDF) [file pone.0222264.s022.pdf]

S14 Table. Differences in the awareness of heart failure symptoms among subgroups (Q14)

Q14: Acute heart failure is a sudden worsening of the signs and symptoms of heart failure. Which of the following is correct for the post-discharge 1-year mortality from acute heart failure?						
	Answer					
	2 in 100 people might die	5 in 100 people might die	10 in 100 people might die	20 in 100 people might die (correct)	Do not know	p-value
Data are presented with %	16.2	25.3	19.6	16.2	22.8	-
Sex						ns
Male	18.0	23.9	20.5	15.9	21.6	
Female	14.3	26.7	18.6	16.5	23.9	
Age (binary)						< 0.001
30-64 years	18.3	25.6	18.3	20.4	17.4	
≥ 65 years	13.9	24.9	20.9	11.7	28.6	
Age (decades)						< 0.001
30-39 years	19.7	27.4	17.2	25.5	10.2	
40-49 years	22.6	24.0	19.2	19.9	14.4	
50-59 years	16.1	25.5	22.4	17.4	18.6	
60-69 years	15.2	30.8	17.0	12.9	24.0	
70-79 years	12.0	18.9	24.6	12.0	32.6	
≥ 80 years	7.7	7.7	19.2	9.6	55.8	
Urbanization level of residence						< 0.001
Urban (*dong*)	17.1	26.8	19.4	16.6	20.1	
Rural (*eup, myeon, ri*)	10.3	15.9	20.7	13.8	39.3	
Educational attainment						< 0.001
Middle school or less	9.7	22.7	19.3	10.1	38.2	
High school	13.6	32.7	18.4	12.9	22.3	
College or more	20.8	21.6	20.4	20.4	16.7	
Do not want to say	0.0	33.3	16.7	25.0	25.0	
Household income (HI, KRW 1,000[*])						< 0.001
HI ≤ 1,000	6.9	9.2	17.2	10.3	56.3	
1,000 < HI ≤ 2,000	16.2	18.9	25.2	14.4	25.2	
2,000 < HI ≤ 3,000	15.7	33.1	15.7	16.1	19.4	
3,000 < HI ≤ 4,000	16.6	25.8	22.7	14.0	21.0	
4,000 < HI ≤ 5,000	17.9	28.8	18.6	17.3	17.3	
HI > 5,000	22.6	23.2	18.3	21.3	14.6	

Do not want to say	2.7	21.6	24.3	21.6	29.7	
Presence of comorbidity†						< 0.01
Yes	13.2	22.2	21.1	14.9	28.7	
No	17.8	26.9	18.8	16.9	19.7	

*US $1=1113.5 Korean won (KRW), October 2018. †Comorbidities (any of hypertension, diabetes, dyslipidemia) of the responders were surveyed.

ns = non-significant.
