# Supplementary material for: Heart failure awareness in the Korean general population: Results from the nationwide survey
Source: PLoS One. 2019 Sep 6;14(9):e0222264. doi: 10.1371/journal.pone.0222264 (PMC6731018; doi:10.1371/journal.pone.0222264)
Supplement: S15 Table — (PDF) [file pone.0222264.s023.pdf]

S15 Table. Differences in the awareness of heart failure symptoms among subgroups (Q15)

Q15: What is the readmission rate within 1 year after discharge from heart failure?						
	Answer					
	2 in 100 people	5 in 100 people	10 in 100 people	20 in 100 people (correct)	Do not know	p-value
Data are presented with %	12.8	24.5	21.5	17.9	23.3	-
Sex						< 0.05
Male	13.0	21.8	24.9	18.8	21.5	
Female	12.5	27.3	18.0	17.1	25.1	
Age (binary)						< 0.001
30-64 years	13.6	24.9	22.4	21.9	17.2	
≥ 65 years	11.9	24.1	20.5	13.7	29.8	
Age (decades)						< 0.001
30-39 years	12.7	29.3	28.7	20.4	8.9	
40-49 years	19.9	24.7	17.8	24.7	13.0	
50-59 years	10.6	23.0	21.7	23.0	21.7	
60-69 years	13.5	27.3	22.6	14.1	22.6	
70-79 years	9.1	21.7	18.9	14.3	36.0	
≥ 80 years	7.7	5.8	11.5	13.5	61.5	
Urbanization level of residence						< 0.001
Urban (*dong*)	14.1	25.8	21.9	17.6	20.6	
Rural (*eup, myeon, ri*)	4.8	16.6	19.3	20.0	39.3	
Educational attainment						< 0.001
Middle school or less	11.6	24.2	12.1	12.6	39.6	
High school	15.5	28.8	17.8	14.6	23.3	
College or more	11.9	22.2	27.6	22.4	15.9	
Do not want to say	0.0	16.7	25.0	8.3	50.0	
Household income (HI, KRW 1,000*)						< 0.001
HI ≤ 1,000	2.3	16.1	6.9	17.2	57.5	
1,000 < HI ≤ 2,000	10.8	23.4	31.5	10.8	23.4	
2,000 < HI ≤ 3,000	14.1	31.0	22.2	10.5	22.2	
3,000 < HI ≤ 4,000	16.2	25.3	24.5	15.3	18.8	
4,000 < HI ≤ 5,000	15.4	25.0	17.9	23.1	18.6	
HI > 5,000	12.8	21.3	23.2	31.1	11.6	
Do not want to say	2.7	10.8	10.8	27.0	48.6	

Presence of comorbidity[†]						< 0.001
Yes	11.2	21.3	17.7	18.5	31.2	
No	13.6	26.2	23.5	17.6	19.1	

[*]US $1=1113.5 Korean won (KRW), October 2018. [†]Comorbidities (any of hypertension, diabetes, dyslipidemia) of the responders were surveyed.

ns = non-significant.
