# Supplementary material for: Heart failure awareness in the Korean general population: Results from the nationwide survey
Source: PLoS One. 2019 Sep 6;14(9):e0222264. doi: 10.1371/journal.pone.0222264 (PMC6731018; doi:10.1371/journal.pone.0222264)
Supplement: S16 Table — (PDF) [file pone.0222264.s024.pdf]

S16 Table. Differences in the awareness of heart failure symptoms among subgroups (Q16)

Q16: What do you think the average healthcare costs per admission are from heart failure? (medical bills that include both out-of-pocket and insurance copayments)							
	Answer						
	1,000,000 KRW*	2,000,000 KRW	3,000,000 KRW	4,000,000 KRW	≥ 5,000,000 KRW (correct)	Do not know	p-value
Data are presented with %	17.6	21.1	20.4	9.4	28.2	3.2	-
Sex							ns
Male	17.6	20.5	17.4	10.5	30.8	3.1	
Female	17.6	21.8	23.5	8.2	25.5	3.3	
Age (binary)							< 0.001
30-64 years	15.7	18.3	20.4	9.3	34.8	1.5	
≥ 65 years	19.7	24.1	20.5	9.5	21.1	5.0	
Age (decades)							< 0.001
30-39 years	12.1	19.1	21.7	7.0	39.5	0.6	
40-49 years	17.1	21.2	20.5	8.9	31.5	0.7	
50-59 years	16.1	16.1	17.4	9.9	37.3	3.1	
60-69 years	17.9	26.1	20.8	8.8	23.5	2.9	
70-79 years	18.3	19.4	23.4	13.1	18.9	6.9	
≥ 80 years	36.5	15.4	13.5	7.7	19.2	7.7	
Urbanization level of residence							< 0.001
Urban (*dong*)	17.1	22.3	21.2	9.6	27.4	2.4	
Rural (*eup, myeon, ri*)	20.7	13.8	15.9	8.3	33.1	8.3	
Educational attainment							< 0.001
Middle school or less	20.8	20.3	25.6	10.1	18.8	4.3	
High school	17.8	29.4	21.4	8.1	20.7	2.6	
College or more	16.3	16.5	17.9	9.9	36.7	2.8	
Do not want to say	16.7	16.7	16.7	8.3	25.0	16.7	
Household income (HI, KRW 1,000*)							< 0.001
HI ≤ 1,000	33.3	10.3	17.2	10.3	21.8	6.9	
1,000 < HI ≤ 2,000	12.6	23.4	19.8	16.2	26.1	1.8	
2,000 < HI ≤ 3,000	19.4	29.4	21.0	8.5	19.8	2.0	
3,000 < HI ≤ 4,000	13.1	21.4	25.3	12.2	26.2	1.7	
4,000 < HI ≤ 5,000	19.2	22.4	16.0	5.1	35.9	1.3	
HI > 5,000	16.5	14.0	20.1	6.1	40.2	3.0	

Do not want to say	10.8	8.1	16.2	8.1	32.4	24.3	
Presence of comorbidity[†]							ns
Yes	18.5	20.8	17.7	11.5	27.2	4.2	
No	17.2	21.3	21.9	8.3	28.7	2.7	

[*]US $1=1113.5 Korean won (KRW), October 2018. [†]Comorbidities (any of hypertension, diabetes, dyslipidemia) of the responders were surveyed.

ns = non-significant.
