# Supplementary material for: Heart failure awareness in the Korean general population: Results from the nationwide survey
Source: PLoS One. 2019 Sep 6;14(9):e0222264. doi: 10.1371/journal.pone.0222264 (PMC6731018; doi:10.1371/journal.pone.0222264)
Supplement: S17 Table — (PDF) [file pone.0222264.s025.pdf]

S17 Table. Differences in the awareness of heart failure symptoms among subgroups (Q17)

Q17: What disease do you think will have the greatest impact on the quality of life? Please select only one.						
	Answer					
	Diabetes	Heart failure	Hypertension	Arthritis	Do not know	p-value
Data are presented with %	36.2	33.8	17.8	10.4	1.7	-
Sex						< 0.05
Male	38.7	34.1	16.3	8.4	2.5	
Female	33.7	33.5	19.4	12.4	1.0	
Age (binary)						< 0.05
30-64 years	35.9	37.8	15.0	9.3	2.1	
≥ 65 years	36.6	29.6	20.9	11.5	1.4	
Age (decades)						< 0.05
30-39 years	38.9	35.7	15.3	8.9	1.3	
40-49 years	34.9	38.4	14.4	11.0	1.4	
50-59 years	36.0	39.1	14.9	6.8	3.1	
60-69 years	31.4	36.4	19.4	11.4	1.5	
70-79 years	40.0	26.9	19.4	11.4	2.3	
≥ 80 years	51.9	5.8	28.8	13.5	0.0	
Urbanization level of residence						< 0.05
Urban (*dong*)	34.8	33.8	19.2	10.6	1.6	
Rural (*eup*, *myeon*, *ri*)	44.8	33.8	9.7	9.0	2.8	
Educational attainment						< 0.001
Middle school or less	41.5	19.3	22.7	15.9	0.5	
High school	34.0	31.4	19.1	13.6	1.9	
College or more	35.9	41.1	14.9	6.2	2.0	
Do not want to say	16.7	41.7	25.0	8.3	8.3	
Household income (HI, KRW 1,000[*])						< 0.001
HI ≤ 1,000	47.1	20.7	18.4	11.5	2.3	
1,000 < HI ≤ 2,000	38.7	27.9	19.8	11.7	1.8	
2,000 < HI ≤ 3,000	36.3	25.4	25.8	11.3	1.2	
3,000 < HI ≤ 4,000	34.9	36.2	18.3	9.2	1.3	
4,000 < HI ≤ 5,000	35.9	39.1	13.5	9.6	1.9	
HI > 5,000	30.5	50.6	7.9	9.8	1.2	
Do not want to say	37.8	27.0	16.2	10.8	8.1	
Presence of comorbidity[†]						ns

Yes	36.8	33.1	16.3	11.5	2.2
No	35.9	34.2	18.6	9.8	1.5

*US $1=1113.5 Korean won (KRW), October 2018. †Comorbidities (any of hypertension, diabetes, dyslipidemia) of the responders were surveyed.

ns = non-significant.
