# Supplementary material for: Heart failure awareness in the Korean general population: Results from the nationwide survey
Source: PLoS One. 2019 Sep 6;14(9):e0222264. doi: 10.1371/journal.pone.0222264 (PMC6731018; doi:10.1371/journal.pone.0222264)
Supplement: S18 Table — (PDF) [file pone.0222264.s026.pdf]

S18 Table. Differences in the awareness of heart failure symptoms among subgroups (Q18)

Q18: If you were a heart failure patient, which of following treatments would you prefer?				
Answer	Treatment that could improve the quality of life	Treatment that allows you to live longer	Cannot decide	p-value
Data are presented with %	70.9	17.6	11.4	-
Sex				ns
Male	72.6	16.7	10.7	
Female	69.2	18.6	12.2	
Age (binary)				< 0.001
30-64 years	76.6	16.6	6.7	
≥ 65 years	64.8	18.7	16.5	
Age (decades)				< 0.001
30-39 years	79.6	17.8	2.5	
40-49 years	75.3	17.8	6.8	
50-59 years	78.9	14.3	6.8	
60-69 years	69.2	18.8	12.0	
70-79 years	61.1	18.9	20.0	
≥ 80 years	51.9	15.4	32.7	
Urbanization level of residence				< 0.01
Urban (*dong*)	72.2	17.8	10.0	
Rural (*eup, myeon, ri*)	63.4	16.6	20.0	
Educational attainment				< 0.001
Middle school or less	52.7	22.7	24.6	
High school	70.2	17.2	12.6	
College or more	79.2	16.1	4.8	
Do not want to say	58.3	8.3	33.3	
Household income (HI, KRW 1,000*)				< 0.001
HI ≤ 1,000	55.2	19.5	25.3	
1,000 < HI ≤ 2,000	64.9	23.4	11.7	
2,000 < HI ≤ 3,000	66.9	20.6	12.5	
3,000 < HI ≤ 4,000	76.0	16.6	7.4	
4,000 < HI ≤ 5,000	71.8	15.4	12.8	
HI > 5,000	84.8	11.6	3.7	
Do not want to say	56.8	18.9	24.3	

Presence of comorbidity†				< 0.05
Yes	67.7	17.4	14.9	
No	72.6	17.8	9.6	

*US $1=1113.5 Korean won (KRW), October 2018. †Comorbidities (any of hypertension, diabetes, dyslipidemia) of the responders were surveyed.

ns = non-significant.
