# Supplementary material for: Heart failure awareness in the Korean general population: Results from the nationwide survey
Source: PLoS One. 2019 Sep 6;14(9):e0222264. doi: 10.1371/journal.pone.0222264 (PMC6731018; doi:10.1371/journal.pone.0222264)
Supplement: S19 Table — (PDF) [file pone.0222264.s027.pdf]

S19 Table. Differences in the awareness of heart failure symptoms among subgroups (Q19)

Q19: Do you agree that 'current heart failure medications could reduce death from heart failure'?				
Answer	Yes	No	Do not know	p-value
Data are presented with %	62.0	14.9	23.1	-
Sex				ns
Male	64.8	14.0	21.3	
Female	59.2	15.9	24.9	
Age (binary)				< 0.05
30-64 years	65.8	12.1	22.1	
≥ 65 years	57.9	17.9	24.1	
Age (decades)				< 0.01
30-39 years	68.8	11.5	19.7	
40-49 years	69.9	13.0	17.1	
50-59 years	62.7	13.0	24.2	
60-69 years	58.4	19.1	22.6	
70-79 years	61.1	14.9	24.0	
≥ 80 years	44.2	9.6	46.2	
Urbanization level of residence				< 0.001
Urban (*dong*)	64.4	15.4	20.2	
Rural (*eup, myeon, ri*)	47.6	11.7	40.7	
Educational attainment				< 0.001
Middle school or less	51.7	16.4	31.9	
High school	59.2	21.0	19.7	
College or more	68.8	10.5	20.6	
Do not want to say	25.0	16.7	58.3	
Household income (HI, KRW 1,000*)				< 0.001
HI ≤ 1,000	47.1	9.2	43.7	
1,000 < HI ≤ 2,000	69.4	15.3	15.3	
2,000 < HI ≤ 3,000	58.5	20.2	21.4	
3,000 < HI ≤ 4,000	65.5	10.9	23.6	
4,000 < HI ≤ 5,000	59.6	17.3	23.1	
HI > 5,000	72.0	12.8	15.2	
Do not want to say	43.2	16.2	40.5	
Presence of comorbidity†				< 0.05
Yes	57.3	18.3	24.4	
No	64.5	13.2	22.3	

*US $1=1113.5 Korean won (KRW), October 2018. †Comorbidities (any of hypertension, diabetes, dyslipidemia) of the responders were

surveyed.

ns = non-significant.
