# Supplementary material for: Heart failure awareness in the Korean general population: Results from the nationwide survey
Source: PLoS One. 2019 Sep 6;14(9):e0222264. doi: 10.1371/journal.pone.0222264 (PMC6731018; doi:10.1371/journal.pone.0222264)
Supplement: S20 Table — (PDF) [file pone.0222264.s028.pdf]

S20 Table. Differences in the awareness of heart failure symptoms among subgroups (Q20)

Q20: Do you agree that 'current heart failure medications could improve the quality of life in patients with heart failure'?				
Answer	Yes	No	Do not know	p-value
Data are presented with %	66.7	14.3	19.0	-
Sex				ns
Male	66.7	15.1	18.2	
Female	66.7	13.5	19.8	
Age (binary)				< 0.001
30-64 years	72.3	11.0	16.6	
≥ 65 years	60.6	17.9	21.5	
Age (decades)				< 0.001
30-39 years	78.3	7.0	14.6	
40-49 years	69.9	13.7	16.4	
50-59 years	69.6	12.4	18.0	
60-69 years	65.7	19.1	15.2	
70-79 years	58.3	16.0	25.7	
≥ 80 years	48.1	7.7	44.2	
Urbanization level of residence				< 0.001
Urban (*dong*)	68.7	15.1	16.2	
Rural (*eup, myeon, ri*)	54.5	9.7	35.9	
Educational attainment				< 0.001
Middle school or less	49.3	17.4	33.3	
High school	62.8	20.4	16.8	
College or more	76.4	9.7	13.9	
Do not want to say	58.3	0.0	41.7	
Household income (HI, KRW 1,000*)				< 0.001
HI ≤ 1,000	48.3	8.0	43.7	
1,000 < HI ≤ 2,000	73.9	11.7	14.4	
2,000 < HI ≤ 3,000	64.1	18.1	17.7	
3,000 < HI ≤ 4,000	70.3	14.0	15.7	
4,000 < HI ≤ 5,000	59.6	20.5	19.9	
HI > 5,000	79.9	9.8	10.4	
Do not want to say	54.1	8.1	37.8	
Presence of comorbidity†				< 0.01
Yes	60.4	17.7	21.9	
No	70.0	12.6	17.5	

*US $1=1113.5 Korean won (KRW), October 2018. †Comorbidities (any of hypertension, diabetes, dyslipidemia) of the responders were

surveyed.

ns = non-significant.
