# Supplementary material for: Heart failure awareness in the Korean general population: Results from the nationwide survey
Source: PLoS One. 2019 Sep 6;14(9):e0222264. doi: 10.1371/journal.pone.0222264 (PMC6731018; doi:10.1371/journal.pone.0222264)
Supplement: S21 Table — (PDF) [file pone.0222264.s029.pdf]

S21 Table. Differences in the awareness of heart failure symptoms among subgroups (Q21)

Q21: Do you agree that ‘current heart failure medications could prevent the occurrence of heart failure’?				
Answer	Yes	No	Do not know	p-value
Data are presented with %	64.9	16.1	19.0	-
Sex				< 0.01
Male	69.2	13.0	17.8	
Female	60.6	19.2	20.2	
Age (binary)				< 0.01
30-64 years	69.2	15.0	15.9	
≥ 65 years	60.4	17.3	22.3	
Age (decades)				< 0.001
30-39 years	76.4	10.8	12.7	
40-49 years	74.0	12.3	13.7	
50-59 years	65.2	18.0	16.8	
60-69 years	61.9	22.0	16.1	
70-79 years	58.3	14.3	27.4	
≥ 80 years	46.2	3.8	50.0	
Urbanization level of residence				< 0.001
Urban (*dong*)	66.4	17.0	16.6	
Rural (*eup, myeon, ri*)	55.9	10.3	33.8	
Educational attainment				< 0.001
Middle school or less	48.8	15.9	35.3	
High school	62.5	21.0	16.5	
College or more	73.2	13.5	13.3	
Do not want to say	58.3	0.0	41.7	
Household income (HI, KRW 1,000*)				< 0.001
HI ≤ 1,000	51.7	5.7	42.5	
1,000 < HI ≤ 2,000	74.8	10.8	14.4	
2,000 < HI ≤ 3,000	58.5	22.6	19.0	
3,000 < HI ≤ 4,000	67.2	17.0	15.7	
4,000 < HI ≤ 5,000	62.2	17.3	20.5	
HI > 5,000	75.6	15.2	9.1	
Do not want to say	59.5	5.4	35.1	
Presence of comorbidity†				ns
Yes	60.1	17.4	22.5	
No	67.5	15.4	17.2	

*US $1=1113.5 Korean won (KRW), October 2018. †Comorbidities (any of hypertension, diabetes, dyslipidemia) of the responders were

surveyed.

ns = non-significant.
