# Supplementary material for: Heart failure awareness in the Korean general population: Results from the nationwide survey
Source: PLoS One. 2019 Sep 6;14(9):e0222264. doi: 10.1371/journal.pone.0222264 (PMC6731018; doi:10.1371/journal.pone.0222264)
Supplement: S22 Table — (PDF) [file pone.0222264.s030.pdf]

S22 Table. Differences in the awareness of heart failure symptoms among subgroups (Q22)

Q22: Suppose one of your friends, colleagues, or neighbors suffers from heart failure. Do you think that they should live quietly and reduce all physical activity?				
Answer	Yes	No	Do not know	p-value
Data are presented with %	59.3	31.6	9.1	-
Sex				< 0.05
Male	61.3	31.8	6.9	
Female	57.3	31.4	11.4	
Age (binary)				< 0.001
30-64 years	57.8	35.9	6.4	
≥ 65 years	61.0	27.0	12.1	
Age (decades)				< 0.001
30-39 years	63.7	33.1	3.2	
40-49 years	54.8	41.1	4.1	
50-59 years	52.8	40.4	6.8	
60-69 years	59.5	30.2	10.3	
70-79 years	66.9	21.7	11.4	
≥ 80 years	51.9	15.4	32.7	
Urbanization level of residence				< 0.01
Urban (*dong*)	61.1	30.8	8.1	
Rural (*eup, myeon, ri*)	48.3	36.6	15.2	
Educational attainment				< 0.001
Middle school or less	58.9	22.7	18.4	
High school	59.2	30.1	10.7	
College or more	59.1	36.7	4.2	
Do not want to say	75.0	8.3	16.7	
Household income (HI, KRW 1,000*)				< 0.001
HI ≤ 1,000	59.8	16.1	24.1	
1,000 < HI ≤ 2,000	65.8	29.7	4.5	
2,000 < HI ≤ 3,000	64.1	25.0	10.9	
3,000 < HI ≤ 4,000	62.9	29.7	7.4	
4,000 < HI ≤ 5,000	49.4	41.7	9.0	
HI > 5,000	51.8	46.3	1.8	
Do not want to say	59.5	21.6	18.9	
Presence of comorbidity†				ns
Yes	58.7	30.1	11.2	

No	59.6	32.4	8.0

*US $1=1113.5 Korean won (KRW), October 2018. †Comorbidities (any of hypertension, diabetes, dyslipidemia) of the responders were surveyed.

ns = non-significant.
