# Supplementary material for: Heart failure awareness in the Korean general population: Results from the nationwide survey
Source: PLoS One. 2019 Sep 6;14(9):e0222264. doi: 10.1371/journal.pone.0222264 (PMC6731018; doi:10.1371/journal.pone.0222264)
Supplement: S23 Table — (PDF) [file pone.0222264.s031.pdf]

S23 Table. Differences in the awareness of heart failure symptoms among subgroups (Q23)

Q23: If you need information about heart failure, where would you visit?						
	Answer					
	Secondary or tertiary care clinic	Primary care clinic	Internet	Oriental medicine clinic	Pharmacy	p-value
Data are presented with %	66.9	19.9	11.6	1.0	0.7	-
Sex						ns
Male	68.6	17.8	12.5	0.8	0.4	
Female	65.1	22.0	10.8	1.2	1.0	
Age (binary)						< 0.001
30-64 years	66.0	14.6	17.8	0.9	0.7	
≥ 65 years	67.8	25.6	5.0	1.0	0.6	
Age (decades)						< 0.001
30-39 years	70.7	12.1	15.9	1.3	0.0	
40-49 years	66.4	10.3	20.5	0.7	2.1	
50-59 years	61.5	16.8	20.5	0.6	0.6	
60-69 years	67.2	22.6	8.2	1.2	0.9	
70-79 years	67.4	29.1	2.3	1.1	0.0	
≥ 80 years	69.2	30.8	0.0	0.0	0.0	
Urbanization level of residence						ns
Urban (*dong*)	66.9	20.1	11.3	1.0	0.8	
Rural (*eup, myeon, ri*)	66.9	18.6	13.8	0.7	0.0	
Educational attainment						< 0.001
Middle school or less	70.0	28.0	0.5	1.0	0.5	
High school	60.8	27.2	9.4	1.3	1.3	
College or more	69.2	12.1	17.7	0.6	0.4	
Do not want to say	66.7	16.7	8.3	8.3	0.0	
Household income (HI, KRW 1,000*)						< 0.001
HI ≤ 1,000	72.4	26.4	1.1	0.0	0.0	
1,000 < HI ≤ 2,000	68.5	25.2	6.3	0.0	0.0	
2,000 < HI ≤ 3,000	71.4	22.2	4.0	0.8	1.6	
3,000 < HI ≤ 4,000	62.9	21.8	14.4	0.9	0.0	
4,000 < HI ≤ 5,000	62.8	15.4	17.3	3.2	1.3	
HI > 5,000	64.6	11.6	23.2	0.0	0.6	
Do not want to say	70.3	16.2	10.8	2.7	0.0	

Presence of comorbidity†						< 0.001
Yes	69.1	24.4	5.1	1.1	0.3	
No	65.7	17.5	15.1	0.9	0.9	

*US $1=1113.5 Korean won (KRW), October 2018. †Comorbidities (any of hypertension, diabetes, dyslipidemia) of the responders were surveyed.

ns = non-significant.
